# Supplementary material for: Tobacco industry and public health responses to state and local efforts to end tobacco sales from 1969-2020
Source: PLoS One. 2020 May 22;15(5):e0233417. doi: 10.1371/journal.pone.0233417 (PMC7244130; doi:10.1371/journal.pone.0233417)
Supplement: S1 Data — (PDF) [file pone.0233417.s001.pdf]

MINUTES

BOARD OF HEALTH

SEPTEMBER 13, 1994

A regular meeting of the Board of Health was held at the Bowditch House, 9 North Street in the City of Salem on Tuesday, September 13, 1994 at 7:00 P.M. Present were George H. Levesque, Chairman; Members Owen Meegan, Esq., Irving Ingraham, MD, Mary Madore, RN, Peter Saindon, Martin Fair, CHO, Health Agent and Leonard Milaszewski. Also present were Joanne Scott, MPH, RS, CHO, Health Agent for the City of Salem and Dr. Leonard A. Dumas, Physician for the Board of Health.

CALL TO ORDER

The regular meeting of September 13, 1994 was called to order at 7:00 P.M.

George Levesque, Chairman welcomed everyone back from a very busy summer and thanked Joanne for the wonderful cook out at her home. The Health Department staff and Board of Health members enjoyed the day.

Mary Madore moved to accept the minutes of the July 12, 1994 regular meeting, seconded by Martin Fair. All in favor.

PROPOSED CIGARETTE VENDING MACHINE REGULATION

Present for discussion were Paul Pechilis, A.P. Vending & Amusement Co., Inc. and Christian D. Vara, Melo-Tone Vending, Inc. Owen Meegan stated the Sub Committee voted not to ban the vending machines but to regulate them with lock-out devises, and recommended the Board accept the Sub Committee's proposed regulation.

George Levesque, Chairman stated the Board and the City Council is in conflict and he will request a Public Hearing.

Paul Pechilis, owner of approximately 8 machines in Salem, stated that Gloucester has not had any violations to date, they work with the Board of Health and the City Council - the lock-out devises have not failed.

Christian Vara, owner of approximately 8 machines in Salem stated he is against selling to minors and is willing to install lock-out devises.

The Board requested they both send a list of the location of their machines to the Health Agent.

Owen Meegan moved to forward the draft regulation to the City Solicitor for his review and legal opinion and a copy to the President of the City Council advising him that there is a conflict between the Board and the City Council regarding the City Ordinance on Cigarette Vending Machines and the Boards' draft regulation that need to be resolved.
Seconded by Leonard Milaszewski. The Board voted 5 in favor and 2 against.

FLU CLINIC

Dr. Dumas spoke briefly about the upcoming Flu Clinic. He stated it would be sometime in November, the state vacccine is late this year. Pneumonia shots are very costly, $8.00 to $10.00 a shot. Joanne Scott stated she would like to have them available.

HILTON STREET SUBDIVISION

Present for discussion were Attorney John Keilty, representing Nancy Singleton, Owner, Jim MacDowell, Eastern Land Survey and Christopher Mello.

A discussion followed regarding the Preliminary/Definitive plans for a proposed subdivision located on Hilton Street.

Dr. Ingraham moved to conditionally approve the Preliminary/Definitive plans for a proposed subdivision on Hilton Street with the Board of Health recommendations
Seconded by Leonard Milaszewski. The Board voted 5 in favor and 2 not voted.

OLDE VILLAGE MALL, 400 HIGHLAND AVENUE

Present for discussion were Attorney Joseph Correnti, representing Rocket Realty Management Co., Hillary Rocket, Owner/Developer and Scott Patrowicz, Engineer.

Attorney Correnti stated Mr. Rocket intends to remove the existing structure and erect a new one, the sewerage will tie into Olde Village Drive, the air conditioners will be installed in the rear of the building.

The Board was very concerned regarding trash, drainage and traffic.
Owen Meegan moved to table issue, more information is needed. Seconded by Leonard Milaszewski. The Board voted 3 in favor, 3 against 1 not voting. Motion failed.

The Board requested Joanne Scott view the site regarding trash/dumpster area.

Peter Saindon moved to notify the Planning Board that the Board of Health is awaiting additional information before making any recommendations.
Seconded by Owen Meegan. The Board voted 4 in favor and 3 against.

STOP & SHOP SUPERMARKET AND JOHN C. JEFFERS
BOSTON STREET AND HOWLEY STREET

At this time the Board recessed. George Levesque stepped down as Chairperson due to his involvement with Stop & Shop. Martin Fair chaired the meeting.

Martin Fair called the meeting back to order at 8:50 p.m.

Present for discussion were Attorney Joseph Correnti of Serafini, Serafini and Darling, representing Stop & Shop and John C. Jeffers, Jim McDowell, Eastern Land Survey, Christopher Mello, Attorney William R. DeMento representing many neighbors, Mary Casey and Mike Averill Grove Street neighbors.

The Board recessed. Martin Fair stepped down as Chairperson.
George Levesque took the chair and called the meeting back to order at 9:45 p.m. and continued the meeting.

Attorney Correnti stated he would be addressing the concerns regarding drainage, trash and traffic. The development would upgrade the site considerably.

The Members were concerned with the noise from diesel trucks, air conditioners and suggested there be a restriction on truck deliveries between 7:00 a.m and 11:00 p.m.

There were questions on storm drain system. Man hole surcharges, not the sanitary sewer system. The sanitary sewer system is in Peabody - does not back up.

Mary Casey expressed her concerns to the Board.

Attorney DeMento asked what has been submitted for this proposal? And what steps has the Health Agent and Board of Health taken?
The Board inquired about the list of concerns that Attorney DeMento was going to submit to the Board. The Board has yet to receive the list. The Board requested Attorney DeMento to submit in writing, all concerns.

Regarding the oil & grease seperator - the Conservation Commission will deal with that issue. The Site drains into the North River. Grove Street is seperate from the proposed site.

Mike Averill expressed his concerns.

The Board advised Ms. Casey, Mr. Averill and Attorney DeMento to list all concerns and submit, in writing the the Health Department.

Owen Meegan moved to table the issue for the Board of Health review the statement and concerns of neighbors or association. Seconded by Leonard Milaszewski. All in favor.

James MacDowell state he would submit his report to the Health Department.

INTERVIEW

Christopher Natale was interviewed for the position of Sanitarian. A discussion took place. Joanne Scott mentioned she had received a resume' from a Karen Springer. Peter Saindon moved to interview Karen Springer, seconded by Mary Madore. All in favor.

MONTHLY REPORTS

The Health Department reports, the Nurse's reports and the TB Clinic reports were accepted as informational.
Leonard Milaszewski moved to accept all the reports, seconded by Dr. Ingraham. All in favor.

Joanne Scott mentioned to the Board, that she will bring the September Chrono file to the next meeting.

ADJOURNMENT

Dr. Ingraham moved to adjourn the meeting, seconded by Mary Madore. All in favor.
The meeting adjourned at 10:30 p.m.

The next regular meeting is scheduled for October 11th at the Bowditch House.

RESPECTFULLY SUBMITTED

Barbara A. Sirois

Barbara A. Sirois
Clerk of the Board of Health

MINUTES

BOARD OF HEALTH

JULY 12, 1994

A regular meeting of the Board of Health was held at the Bowditch House, 9 North Street in the City of Salem on Tuesday, July 12,1994 at 7:00 P.M. Present were George H. Levesque, Chairman; Members Owen Meegan, Esq., Irving Ingraham, MD and Mary Madore, RN Also present were Joanne Scott, MPH, RS, CHO, Health Agent and Councillor Leonard F. O'Leary, Liaison for the Public Health, Safety & Environment Committee. Peter Saindon was absent, Martin Fair, CHO, Health Agent and Leonard Milaszewski were on vacation.

CALL TO ORDER

The regular meeting of July 12, 1994 was called to order at 7:15 P.M.

Owen Meegan moved to accept the minutes of the May 17th regular meeting, seconded by Mary Madore. All in favor.

Mary Madore moved to accept the minutes of the June 14th regular meeting and the June 28th special meeting, seconded by Owen Meegan. All in favor.

In reference to the Smoking Issue, Owen Meegan, Chairman of Sub Committee on Cigarette Vending Machines moved the Board send his report/draft to the City Council in answer to the City Council's request. Seconded by Mary Madore. All in favor.

APPLICATION FOR MASSAGE

Present for discussion were Alex Panos, Owner of Image Concepts, 12 Front Street in the Central Development District (B-5), Attorney Carl D. Goodman and Natalie Chiavoli, Massage Therapist. Attorney Goodman reviewed the 2 issues that concerned the Board.

#1 - Zoning - a letter received from Leo Tremblay, Building Inspector states massage therapy is a legal use and permitted in the Central Development District (B-5).

#2 - Liability if the Board waives the requirement of a Physical Therapist on site - a letter received from Robert Ledoux, City Solicitor states" the Board should expect that future applicants would request and expect the same waiver". "If the Board is inclined to waive the requirement of a physical therapist, I see no reason why the requirement should not be stricken from Regulation 11 entirely".

A discussion followed. George Levesque, Chairman requested the Board not grant the license, he would prefer a physical therapist on site. Joanne Scott, Health Agent voiced her concern about the hand washing sink not being in the same room. Dr. Ingraham felt there was no danger to having the hand washing sink across the hall. Mary Madore agreed with the Doctor.

Owen Meegan moved that the application of Alex' Place, Inc., d.b.a. Alex & Lucille Image Concepts, TO CONDUCT AN ESTABLISHMENT FOR GIVING OF BODY MASSAGE, be approved and that the applicant be granted exceptions from the provisions of Regulation 11 as follows:

5.D. Allow use of washing facilities in restroom located in close proximity to massage therapy room.

5.S. Massage Theapist may practice on clients of either sex.

5.Y. No licensed Physical Therapist will be employed.

Dr. Ingraham seconded the motion. All in favor.

REVIEW OF PROJECT RAP CONTRACT

Present for discussion was Kim Wilkins, Representative from Rap. Ms. Wilkins presented a very informative report. The Board thanked Ms. Wilkins for taking the time to attend the meeting and for the report.

REVIEW OF BASS RIVER CONTRACT

Present for discussion was Edward A. Potvin, Executive Director. Mr. Potvin presented a very informative report and thanked the Board for 16 years of support and hoped for many more. The Board thanked Mr. Potvin for taking the time to attend the meeting and for the report.

MONTHLY REPORTS

The Health Department reports, and the Nurses reports were accepted as informational.

Councillor O'Leary requested the drinking water at Bakers Island be tested.

Joanne Scott, Health Agent informed the Board that Jeffrey Vaughan, Sanitarian is now working in the Health Department. Dawn Marie Cameron, RN, Public Health Nurse will begin her employment on August 1, 1994.

Ms. Scott also requested a weeks vacation to be taken the last week of August.
Owen Meegan moved to accept the request and advance vacation pay to Ms. Scott. Mary Madore seconded the motion. All in favor.

ADJOURNMENT

Owen Meegan moved to adjourn the meeting. Mary Madore seconded the motion. All in favor. The meeting adjourned at 9:00 p.m.

George Levesque, Chairman informed the Board that the August meeting will be cancelled.

Next regular meeting scheduled September 13, 1994 at the Bowditch House.

RESPECTFULLY SUBMITTED

Barbara A. Sirois

Barbara A. Sirois
Clerk of the Board of Health

MINUTES

BOARD OF HEALTH

JUNE 14, 1994

A regular meeting of the Board of Health was held at the Salem High School Library, Willson Street in the City of Salem on Tuesday, June 14, 1994 at 7:00 P.M. Present were George H. Levesque, Chairman; Members Peter Saindon, Owen Meegan, Martin Fair, Leonard Milaszewski and Mary Madore. Also present was Joanne Scott, Health Agent.

CALL TO ORDER

The regular meeting of June 14, 1994 was called to order at 7:15 P.M.

At this time Acting Chairman Peter Saindon tabled the minutes of the May 17, 1994 meeting. The Chairman, George Levesque is away on a well deserved vacation.

SMOKING UPDATE

Owen Meegan, Chairman of Sub Committee on Cigarette Vending Machines reviewed the draft that was presented to the Board. He stated the City of Salem Ordinance has no enforcement and no fines. The Board of Health should draw up a regulation that is more stringent. Joanne Scott, Health Agent, stated the Board has to take a position on this issue, we must "educate and enforce" there are plenty of regulations and laws on the books already.
Owen Meegan suggested that a model regulation should be drafted regarding vending machines and lock-out devices.

PRELIMINARY PLAN REVIEW COLBY STREET

Present for discussion was Attorney Joseph Correnti representing Salem Hospital to construct a building for a childcare facility at 1-3 Colby Street in the City of Salem.

Attorney Correnti presented the preliminary plan review and stated a Public Hearing will be held at a later date.
The Board accepted the presentation as informational.

INTERVIEWS

Interviewed for the Position of Public Health Nurse were; Tanis Kahn and Dawn Marie Cameron.

Interviewed for the Position of Sanitarian was; Martin Plecinoga.

PROPOSED RESIDENTIAL KITCHEN - 8 RANDALL STREET

Present for discussion were Elizabeth and Andre M. Aoun applying for a permit to operate a residential kitchen at their property located at 8 Randall Street in the City of Salem.

Leonard Milaszewski moved to approve the plan. Owen Meegan seconded the motion. A vote was taken 3 in favor and 1 against (Martin Fair).

MONTHLY REPORTS

The Health Department reports, the Nurses report and the TB Clinic reports were accepted as informational.

ADJOURNMENT

Leonard Milaszewski moved to adjourn the meeting at 11:00 P.M. Owen seconded the motion. All in favor.

Next regular meeting scheduled July 12, 1994 at the Bowditch House.

RESPECTFULLY SUBMITTED

Barbara A. Sirois

Barbara A. Sirois
Clerk of the Board of Health

MINUTES

BOARD OF HEALTH

May 17, 1994

A regular meeting of the Board of Health was held at the Salem High School Library, Willson Street in the City of Salem on Tuesday, May 17, 1994 at 7:00 P. M. Present were George H. Levesque, Chairman; Members; Peter Saindon, Owen Meegan and New Member Mary MadorE. Also present were Joanne Scott, Health Agent, Dr. Leonard Dumas, Physician for the Board of Health and Councillor Leonard F. O'Leary, Liaison for the Public Health Committee.

CALL TO ORDER

The regular meeting of May 17, 1994 was called to order at 7:15 p.m.

At this time the Board of Health Chairman and members congratulated Mary Madore on her appointment as a member of the Board of Health. Mary was warmly welcomed by all.

Owen Meegan moved to accept the minutes of the April 12, 1994 regular meeting and the May 3, 1994 special meeting. Seconded by Peter Saindon. All in favor.

NORTH SHORE PULMONARY CLINIC

Present for discussion was Mary Jane Thomas, Clerk at the Pulmonary Clinic located at Salem Hospital. The Board welcomed Mary Jane and inquired about her duties. The Salem Health Department is mandated by the State to provide clerical assistance at the Clinic. Mary Jane explained the position is part-time, 18 hours per week, the paperwork has increased due to the increase of TB patients in the area. The Clinic provides TB shots to patients, follow up medications and appointments and there has been 2 new cases in the past two weeks.

Mary Jane also requested 2 weeks vacation this year without pay, due to her family moving to a new home. Joanne Scott, Health Agent stated Sue Kocur, Clerk in the Health Department could fill in for Mary Jane at the clinic for the 2 weeks. Mary Jane has been employed for approximately 6 years and has never requested vacation etc. George Levesque, Chairman suggested Mary Jane receive pay if at all possible.

Owen Meegan moved contingent upon Personnel, 2 weeks paid vacation be granted to Mary Jane. Peter Saindon seconded the motion. All in favor.

APPLICATION FOR MASSAGE

Present for discussion were Attorney Carl D. Goodman and Alex Panos, owner of Image Concepts, 12 Front Street in the City of Salem.

Attorney Goodman presented a new plan showing the lay out of the establishment. Joanne Scott, Health Agent reviewed the applications and the inspection report of the establishment for massage along with the requirements to be licensed to practice massage.

A letter received from Attorney Goodman was reviewed regarding the Zoning Ordinance in the City of Salem. The Business in question Image Concepts located at 12 Front Street is located in a B-5 District which is sometimes known as the Central Development District was stated in the letter. A discussion followed.

Councillor O'Leary was concerned and suggested the Board receive a legal opinion from the City Solicitor. Joanne Scott, Health Agent asked Attorney Goodman iF there would be a Physical Therapist on the premesis ? The answer was no.

Mary Madore questioned the liability of not having a Physical Therapist on the premises. Peter Saindon suggested sending information regarding liability and zoning to be reviewed by the City Solicitor.

Owen Meegan moved to request the legal opinion of the City Solicitor as to the Zoning issues operating a Massage Therapist Business at 12 Front Street, and his opinion to the liability without a Physical Therapist.
Mary Madore seconded the motion. The Board voted 2 for and 2 against.

Owen Meegan moved to table the issue for the next meeting in June.
Peter Saindon seconded the motion. All in favor.

SMOKING ISSUES - REVIEW OF CURRENT AND MODEL REGULATIONS, LAWS, REPRINTS AND CORRESPONDENCE

Present for discussion were Robert Ledoux, City Solicitor, many Restaurant owners and concerned citizens.

Mr. Ledoux stated the City of Salem can prohibit cigarette vending machines and establishments can be fined a maximum of $300 if a minor is allowed to purchase from such machines.

A discussion followed with many questions being answered by Solicitor Ledoux. He stated the Board of Health can control the license of vending machines. It is discrimination to allow the machines in private clubs and not in restaurants and bars. There is no difference if the machine is rented or owned by the establishment.

Councillor O'Leary stated the City Ordinance mentioned no fine and no enforcement. Solicitor Ledoux suggesed the Police Department can enforce and the Board of Health can reword the ordinance regarding the fines and enforcement.

George Levesque, Chairman stated the goal for the Board of Health is to write a regulation and follow up on the enforcement. Chairman Levesque appointed a Sub Committee with Owen Meegan as Chairman and members Mary Madore and Dr. Ingraham.

SALEM SOUND 2000

Present for discussion was Sam Cleaves, Monitoring Coordinator SSASI, (Shoreline Survey and Source Indentification). Mr. Cleaves explained to the Board that Salem Sound began in 1991, and is a consortium of residents, business people, government agencies and marine professionals concerned with the water quality and the future of Salem Sound. It is within Massachusetts Bay from Manchester-by-the-Sea to Marblehead, with the assistance of a grant from th Massachusetts Bays Program.

The Primary concern is pollution from storm drain runoff, illegal discharges or sewer connections, failing septic systems etc. There are many volunteers involved in the program.

Joanne Scott, Health Agent mentioned to Mr. Cleaves the role the Health Department takes in sampling the waters at area beaches every other week during the summer months. Ms. Scott stated she would forward a list of area beaches and the test results to Mr. Cleaves.

The Board accepted this report as informational.

MONTHLY REPORTS

The Health Department reports, the Nurses report and the TB Clinic report were accepted as informational.

ADJOURNMENT

Owen Meegan moved to adjourn the meeting at 9:30 p.m. Seconded by Peter Saindon. All in favor.

Next regular meeting scheduled June 14, 1994

RESPECTFULLY SUBMITTED

Barbara A. Sirois

Barbara A. Sirois
Clerk of the Board of Health

MINUTES

BOARD OF HEALTH

May 3, 1994

A special meeting of the Board of Health was held at the Bowditch House, 9 North Street in the City of Salem on Tuesday, May 3, 1994 at 7:00 P. M. Present were George H. Levesque, Chairman; Members; Peter Saindon, Martin Fair, Owen Meegan and Irving Ingraham, MD. Also present was Joanne Scott, MPH, RS, CHO, Health Agent.

CALL TO ORDER

The special meeting of May 3, 1994 was called to order at 7:20 p.m.

The special meeting was held to conduct an interview for the position of Senior Sanitarian. Civil Service was contacted regarding an updated list. There is no list.

Virginia Moustakis, presently a Sanitarian, was the only in house candidate. A discussion took place. Dr Ingraham moved to hire Virginia Moustakis as the Senior Sanitarian. Seconded by Peter Saindon. All in favor.

Peter Saindon moved for immediate reconsideration in the hopes it would not prevail. Seconded by Dr. Ingraham. So voted.

Peter Saindon moved to adjourn the meeting at 8:15 p.m. Seconded by Owen Meegan. All in favor.

Next regular meeting scheduled for May 17, 1994

RESPECTFULLY SUBMITTED BY

Barbara A. Sirois

Barbara A. Sirois
Clerk of the Board of Health

MINUTES

BOARD OF HEALTH

April 12, 1994

A regular meeting of the Board of Health was held at the Bowditch House, 9 North Street in the City of Salem on Tuesday, April 12, 1994 at 7:00 P. M. Present were George H. Levesque, Chairman; Members; Peter Saindon, Martin Fair, Owen Meegan, Irving Ingraham, MD. and Leonard Milaszewski. Also present were Joanne Scott, MPH, RS, CHO, Health Agent, Dr. Leonard Dumas, Physician for the Board of Health and Councillor Leonard F. O'Leary, Liaison for the Public Health Committee.

CALL TO ORDER

The regular meeting of April 12, 1994 was called to order at 7:05 p.m.

At this time the Board of Health Chairman and members congratulated Joanne Scott on her new position as Health Agent Joanne was warmly welcomed by all.

Leonard Milaszewski moved to accept the minutes of the March 8,1994 regular meeting. Seconded by Peter Saindon. All in favor.

The monthly reports were discussed and accepted as informative.
Leonard Milaszewski moved to invite Mary Jane Thomas, part - time clerk at the Pulmonary Clinic to the May 10, 1994 meeting. Seconded by Owen Meegan. So voted.

Martin Fair moved to receive and place on file the following: letter of congratulations to Joanne Scott, Health Agent and letters of resignation from Julie Forsberg, Sanitarian, Lori Silva, RN Public Health Nurse and Gene Collins, Board of Health member.

The Board requested letters of appreciation be sent to Julie, Lori and Gene.

The Public Health Nurses' position was discussed. Tracy Giarla, LPN is currently filling the position on a part-time basis and doing an excellent job. Joanne Scott, Health Agent stated a letter was sent to the Mayor requesting that the position stay in the Health Department, rather than being contracted out. She asked the Board for their support.
Owen Meegan moved to keep the position in the Health Department and support the Health Agent 100%. Seconded by Leonard Milaszewski. The Board voted unanimously in favor.

A discussion regarding the vacant positions in the Health Department took place. The Board requested Joanne Scott, Health Agent to notify Personnel to post the Senior Sanitarian, Sanitarian and Public Health Nurse positions as soon as possible and request a Civil Service list for all positions. The Board also requested the three job descriptions be revised.

A discussion regarding a larger room for Board of Health meetings took place. Suggestions were Police Station Auditorium, 1 Salem Green or Salem High School. Joanne Scott, Health Agent will investigate the possibilities for the next meeting.

Correspondence was received by GEI Consultants, Inc. in regards to field work to take place at the Salem Acres Superfund Site off Barcelona Avenue in the month of April. George Levesque, Chairman will go to the site next week. The Board accepted this as informative.

MUSCULAR THERAPY
NATALIE CHIAVOLI'S RESUME AND EXPERIENCE

Present for discussion were Carl D. Goodman, Attorney at Law, Alex and Lucille Panos, Owners of Alex and Lucille's Image Concepts, 12 Front Street, Salem, MA, Natalie Chiavoli, Muscular Therapist and Dr. Lewis Hayes.

All necessary documents have been received by the Board of Health. Attorney Goodman stated Alex and Lucille Panos are seeking a Massage License for both the establishment and the individual. Their business has been in Salem since 1962 and at the present site since 1977, and has grown extensively. Services include hair salon, facials, manicures,cosmotology, image consulting and would like to include massage/muscular therapy.

Natalie Chiavoli is licensed in Swampscott, Danvers and New Hampshire and is highly qualified to practice massage. Ms. Chiavoli works with many Doctors in Danvers and has many referrals from Doctors and Physical Therapists.

Dr. Hayes, North Shore Medical Center stated he has known Ms. Chiavoli for 11 years and has referred many of his patients to her. Muscular Therapy is deep and theraputic and is very effective in battered women etc. He recommends Ms. Chiavoli highly.

The service would be conducted on the 2nd floor of the building, The Building Inspector has approved the area. The Health Department has not inspected the site as of this date. The rest rooms are not in the room. The area is zoned for business.

George Levesque, Chairman suggested the Health Agent along with a Sanitarian inspect the area in question. The issued was tabled until the meeting.

CIGARETTE VENDING MACHINES

Present for discussion were Paul Harrington, Teacher of Health and Biology at Salem High School, and students. Also present were David Collins Director of the Tobacco Control Program, Mark Levine, Melo-ToneVending, Inc. and other concerned citizens.

A discussion took place. Mr. Harrington stated smoking is a definite health problem and students must be educated and made aware. At Salem High 35% of the students smoking are purchasing their cigarettes from vending machines. He suggests banning the machines totally from the City of Salem.

The concensus of the meeting on the whole was to ban vending machines everywhere except the Bars. If cigarettes are sold to minors (under 18) the establishment will be fined. Lock out devises are not effective.

Bill O'Keefe, Bartender-Ward II Social Club stated vending machines must be in view of the bartender. Banning machines will not work, if teens want cigarettes they will manage to get them, he suggests increasing the fine from $100.00 to $1000.00.

Mark Levine, Melo Tone Vending, Inc. stated he is in support of regulating tobacco sales to minors. He has no control once the machine is in the bar. Mr. Levine demonstrated the lock out devise on a vending machine. Everyone has to take responsibility, if lock our is not working properly the vendor is responsible. The public must be educated. There are 12 to 15 vending machines currently in the City. He suggested the Board consider the option of the lock out devise.

The Board recessed at 9:15 p.m.

The meeting came back to order at 9:30 p.m.

The Board of Health and Health Agent expressed their opinions. Owen Meegan moved to explore the issue of licensing vending machines, increasing the fine to $1000.00 for violation of sales to minors and and outright ban of sales of cigarettes in the City of Salem.
Seconded by Leonard Milaszewski. 4 in favor 1 against, Peter Saindon.

Owen Meegan moved to table the issue until the next meeting. Seconded by Leonard Milaszewski. So voted.

PRELIMINARY SUBDIVISION PLAN
MARKET PLACE
SALEM AND PEABODY MASSACHUSETTS

Present for discussion were Attorney John R. Serafini, Sr. and Jim McDowell, Project Manager. Mr. McDowell stated it was a revised pre subdivision plan (form B) divided into lots. The definitive plan (form C) will be presented at a later date.

The Board reviewed the plan presented and await the Definitive Plan.

ADJOURNMENT

Leonard Milaszewski moved to adjourn the meeting at 10:30 p.m. Seconded by Owen Meegan. All in favor.

Next regular meeting scheduled May 10, 1994

RESPECTFULLY SUBMITTED

Barbara A. Sirois

Barbara A. Sirois
Clerk of the Board of Health

MINUTES

BOARD OF HEALTH

March 8, 1994

A regular meeting of the Board of Health was held at the Bowditch House, 9 North Street in the City of Salem on Tuesday, March 8, 1994 at 7:00 P. M. Present were George H. Levesque, Chairman; Members; Peter Saindon, Irving Ingraham, Gene Collins and Owen Meegan. Also present were Robert E. Blenkhorn, CHO, Health Agent, Dr. Leonard Dumas, Physician for the Board of Health and Councillor Leonard F. O'Leary Liaison for the Public Health Committee.

Mr. Blenkhorn introduced David Collins, Director of the Tobacco Control Program to the Board. The Tobacco Control Program (TCP) is a division of the Health Department.

The February 15th meeting was continued from last month. Monthly reports were accepted as informational.

Gene Collins moved to send a letter to the Park Commission thanking them for the use of their Board room for Board of Health monthly meetings.
Peter Saindon seconded the motion. All in favor.

Gene Collins moved to adjourn the February 15th meeting at 7:30 p.m.
Peter Saindon seconded the motion. All in favor.

CALL TO ORDER

The regular meeting of March 8, 1994 was called to order at 7:35 p.m.

Owen Meegan moved to accept the minutes of the February 15, 1994 regular meeting. Seconded by Gene Collins. All in favor.

Peter Saindon suggested the time of adjournment be recorded in the minutes.

Owen Meegan commented on the inspectors report regarding restaurant inspections.

OTHER BUSINESS

David Collins, Director of the Tobacco Control Program (TCP) addressed the Board. The North Shore Area Boards of Health Collaborative (NSABOHC) was awarded a $300,000.00 Grant, hence the Tobacco Control Program was formed. The program will consist of 3 positions, Director, Administrative Assistant and Community Educator. David is in the process of filling the other positions.

The 8 communities involved are Salem (the host City), Lynn, Danvers, Saugus, Marblehead, Swampscott, Nahant and Peabody. One of the first steps is zeroing in to what the needs are in the Community, adopting a regulation on Cigarette Vending Machines in the City. Together with the Board of Health the Tobacco Control Program's success will be accomplished.

The discussion regarding this program will continue at the next Board of Health meeting, April 12, 1994.

North Beverly Environment Action Committee report was received and put on file.

The Board held a discussion regarding the Health Department Staff and the new Health Agent. The Board suggested the sanitarians start the process of restaurant inspection in a professional and timely fashion.

MONTHLY REPORTS

The Health Department Reports, were accepted as informational.
It was noted that Lori Silva, RN, Public Health Nurse is on vacation February 25th thru March 9, 1994 off to Italy. She will include the February stats in the March report.

At this time George Levesque, Chairman wished Mr. Blenkhorn good luck on his retirement and thanked him for his loyal service.

Mr. Blenkhorn addressed the Board, stating it has been an honor to work with this Board and past Boards, the staff past and present and if the Board wishes, he would be glad to stay on as Health Agent until the new Agent starts.

Peter Saindon moved to keep Mr. Blenkhorn as a consultant to the Board of Health for not more than 30 days. Owen Meegan seconded the motion. All in favor.

ADJOURNMENT

Owen Meegan moved to adjourn the meeting at 8:30 p.m. Peter Saindon seconded the motion. All in favor.

Next regular meeting scheduled April 12, 1994

RESPECTFULLY SUBMITTED

Barbara A. Sirois

Barbara A. Sirois
Clerk of the Board
