# Supplementary material for: Tobacco industry and public health responses to state and local efforts to end tobacco sales from 1969-2020
Source: PLoS One. 2020 May 22;15(5):e0233417. doi: 10.1371/journal.pone.0233417 (PMC7244130; doi:10.1371/journal.pone.0233417)
Supplement: S2 Data — (PDF) [file pone.0233417.s002.pdf]

Peter

GRAY TOWN COUNCIL
DECEMBER 1, 1998 AGENDA
7:00P.M. - STIMSON HALL

Preceding the December 1, 1998 Town Council Meeting. The Gray Town Council shall hold a Public Hearing on Application for Liquor License for Richard F. Prince for The American Legion, The Gray Post No. 86, Inc. located on Lewiston Road.

Preceding the December 1, 1998 Town Council Meeting. The Gray Town Council Public Hearing on Application for Liquor License for Daniel W. Gilliland Sr. for Pizza Paddle Restaurant and Portland North Banquet Center located at Gray Plaza.

First Order of Business:

Approval of the October 6th, October 20, and November 10th, and November 17th Town Council Minutes. postponed

Second Order of Business:

Reports from the Council Chair
Reports from the Town Manager — No Yarmouth walk town line 15
Committee Reports
Council Correspondence

Third Order of Business:

Committee Appointments and Resignations:
Committee Business
Council Business

Fourth Order of Business:

Reading of and Additions to the Agenda

Fifth Order of Business:

✓ Order 198: Council discussion with Terry Brooks United States Postal Service regarding future of The United States Postal Facility.

GRAY TOWN COUNCIL
DECEMBER 1, 1998 AGENDA
7:00P.M. - STIMSON HALL

Order 199: Council review and action on the application for Liquor License for Richard E. Prince for The American Legion, The Gray Post No 86, Inc. located on Lewiston Rd. 5-0

This order requires a majority vote after an opportunity for public comment.

Order 200: Council review and action on the application for Liquor License for Daniel W. Gilliland Sr. for The Pizza Paddle and Portland North Banquet Center. 5-0

This order requires a majority vote after an opportunity for public comment.

Order 201: Council review and action on opening election poles at 7:00am. Sponsor Counciler Monroe Natural

This order requires a majority vote after an opportunity for public comment.

Order 202: Council review and action to expend municipal operational funds on pending public easement, Notched Pond Estates, for 1998-99 snow plowing and sanding services. Presented by the Town Manager. NO second

This order requires a majority vote an opportunity for public comment.

Order 203: Council review and action pursuant to 22 M.R.S.A. & 1556, that the Town Clerk be and hereby is authorized and directed to sen notice to the last known address of each retail tobacco licensee doing business in Gray that the Town Council will hold a public hearing on the first reading of an ordinance to prohibit the sale of tobacco products in Gray on the _____ day of ____________, 1998 at 7:00p.m., at Stimson Hall.

1st 2nd 3rd Tues
5th, 12th, 19th

GRAY TOWN COUNCIL
DECEMBER 1, 1998 AGENDA
7:00P.M. - STIMSON HALL

Order 204: Council discussion and action concerning Councilor Cook regarding Town Council Rule 602.23, which states:

Council Voting:

Every member present when a question is put shall give his/her vote unless the council, for special reasons, shall excuse him/her. Applications to be so excused must be made before the Council is divided, or before the calling of the yea's and nays, and decided without debate.

Order 205: Executive session reg arding personnel issue.

Sixth Order of Business

Tabled Orders: Council review and action on acceptance of "Cobb Crossing" as a town way. Submitted by Town Manager. (Cobb Crossing is located in the May Meadow subdivision)

Standing Orders:

Order 010: Council approval of Pole Permits for Central Maine Power and Pine Tree Telephone Company.

This order requires a majority vote after an opportunity for public comment.

Order 011: Public Discussion of Non-Agenda Items.

Order 012: Council approval of Concealed Firearms Permits, as requested by the Maine State Police Licensing Division.

This order requires a majority vote after an opportunity for public comment.

Seventh Order of Business: Adjournment

M. Tose

Gray Town Council Meeting
December 1, 1998

Present:	Town Council Members – Chair Mark A. Sanborn, Vice Chair Anthony V. Cook, Frances Monroe, Ronald Norton, and Jack Goosetrey
Also Present:	Town Manager Peter Jankowski, Recorder Jan Bourret
Others:	Terry Brooks, Daniel Gilliland, Allan Pettle, Mike Berrard, Davene Dadiego, Cal Cutter, Julie McCale, Cathleen Manchester, Stephen Amergian, Donald Whitney, Brad Fogg and many other residents of the Town of Gray.

Chair called the meeting of the Gray Town Council to order at 7:00 p.m.

Preceding the December 1, 1998 Town Council Meeting, The Gray Town Council shall hold a Public Hearing of Application for Liquor License for Richard F. Prince for the American Legion, Gray Post No. 86, Inc., located on Lewiston Road.

With no comments from the Council or the public, the public hearing was closed.

Preceding the December 1, 1998 Town Council Meeting, the Gray Town Council will hold a Public Hearing on the Application for a Liquor License for Daniel W. Gilliland Sr. for Pizza Paddle Restaurant and Portland North Banquet Center located at Gray Plaza.

With no comments from the Council or the public, the public hearing was closed.

First Order of Business
Approval of the October 6th, October 20th, November 10th and November 17th 1998 Town Council Minutes

Chair Sanborn postponed the approval of the minutes until the next meeting, giving more time for the Council to review the minutes submitted by the new recorder.

Councilor Monroe asked that the new recorder include all motions in the meeting minutes.

The Council voted unanimously to postpone.

Second Order of Business
Reports from the Chair

Chair Sanborn extended his thanks to both Gray Fire and Rescue and the Historical Society for the outstanding work done in the center of town for the annual Christmas tree lighting.

Reports from the Town Manager
None

Committee Reports
None

Council Correspondence
None

Committee Correspondence
None

Third Order of Business
Committee Appointments and Resignations
None

Committee Business
None

Council Business
None

Fourth Order of Business
Reading of and Additions to the Agenda
None

Fifth Order of Business

Order 198: Council discussion with Terry Brooks, United States Postal Service, regarding future of the United States postal facility.

Terry Brooks addressed the Councilors to discuss the upcoming plans for a postal facility in the Town of Gray. The first step is to review the existing facility and determine if it could be expanded and if by doing so this would be the best solution. The current post office is approximately 2300 square feet and parking space may be an issue if expansion is considered, since the lot is only 75 feet wide. Mr. Brooks said that he was looking to the Town to see if there is a preferred area for the new facility. We solicit proposals and wait for the town to review and discuss these proposals. There is a 15-day waiting period for this review. After that time, we submit our request and the town has a 30-day period for review. If a decision is made that the town is not happy with, then there are three opportunities for appeal. If the decision to build rather than expand was to be proposed, that could be appealed. If the location that is selected is not one that the town council or an individual citizen likes, an appeal is available at that point as well. Lastly, an appeal is available to the Town Council (not individual citizens) after the site is determined. Mr. Brooks requested that during the next 15-day period any Council members who had questions could contact him, the Postmaster or Mr. Desjardin.

Chair Sanborn asked if the Council had any questions for Mr. Brooks. Councilor Monroe wanted to know how far into the future are postal facilities planned for. Mr. Brooks explained that the site is planned for a twenty-year period and the facility for a ten-year period. Councilor Monroe asked if there was a possibility of two postal locations in the town. Mr. Brooks said that there were no plans for that at the present time.

Councilor Norton asked what the present size of the post office was and when the target date was for completion of the new facility. Mr. Brooks stated that the current post office was 2300

square feet. The process requires a minimum of 145 days (in light of the appeals process). The project expects to be completed around Christmas 1999.

Councilor Sanborn inquired if Mr. Brooks had begun looking at land and possible site locations. Mr. Brooks said that he had had some calls from brokers in Portland that wanted to work with him, but he informed them that he preferred going to the community first with suggestions. Also, the owner of the shopping center on Route 26 had called about some land. The space needs at that location seemed to be adequate, but he was unsure if there was enough land available to sell.

Councilor Monroe said that the senior citizens in the Town walk to the current post office daily. It is a concern that these citizens continue to have access to their postal facility. Mr. Brooks explained that most often they try to keep the new location within a certain distance of the existing one. This poses more of a problem in rural areas.

Mr. Sanborn thanked Terry Brooks for his presentation and suggested to the Council that the Economic Development Committee looks at this issue and that is discussed at the next workshop.

Order 199: Council review and action on the application for Liquor License for Richard E., Prince for the American Legion, Gray Post No. 86, Inc. located on Lewiston Road

It was moved by Mr. Cook, seconded by Mr. Norton to review the application for Richard Prince. There was no further discussion or comment. The Council vote was unanimous.

Order 200: Council review and action on the application for Liquor License for Daniel W. Gilliland Sr. for the Pizza Paddle and Portland North Banquet Center.

It was moved by Mr. Cook, seconded by Ms. Monroe to review the application for Mr. Gilliland. There was no further discussion or comment. The Council vote was unanimous.

Order 201: Council review and action on opening election poles at 7:00 am. Sponsored by Councilor Monroe.

It was moved by Councilor Monroe that the Council authorize opening election poles at 7:00 a.m. Councilor Norton seconded.

Mr. Cook made an amendment to the motion that on national election voting days, the polls open at 6:00 a.m. Mr. Norton seconded. All Council members were in favor of the main motion and the amended motion also carried unanimously.

Order 202: Council review and action to expend municipal operational funds on pending public easement, Notched Pond Estates, for 1998-1999 snow plowing and sanding services. Presented by the Town Manager.

Allan Pettle, of 181 Warren Shores, addressed the Council. He currently resides at Notched Pond Estates. Mr. Pettle explained that his home is on the waterfront in Gray, but the physical buildings, due to restrictions to the lot for building, are in New Gloucester.

Council Cook mentioned that he had spoken with the Public Works Director and that there needed to be four homes on a road before the Town of Gray would provide plowing and sanding. It was the Public Works Directors understanding that there were only two residences on the road at the present time. Mr. Pettle said that there were four homes on the road. The Pettle's, Mr. Campbell's and Mr. Chamberlain's homes were on the road. He explained that Mr. Campbell's residence was located in both Gray and New Gloucester. Mr. Pettle stated that the road was approved by the Town of Gray in 1987 and many tax dollars have been paid to the Town over the years. Many more people planned to be building there in the future, as well.

Councilor Monroe inquired if the Town Manager was aware of where the tax dollars were paid by the residents on that road. Mr. Jankowski did not have that information but added that anyone who comes to the Town for plowing and sanding are put on the agenda.

There was no motion made by the Council on this item.

Order 203: Council review and action pursuant to 22 MRSA & 1556, that the Town Clerk be and hereby is authorized and directed to send notice to the last know address of each retail tobacco licensee doing business in Gray that the Town Council will hold a public hearing on the first reading of an ordinance to prohibit the sale of tobacco products in gray on the _____ day of ____________, 1998, at 7:00 p.m. at Stimson Hall.
Sponsored by Chair Sanborn

Councilor Sanborn made the motion for this order with Councilor Norton seconding.

The Chair explained that he wanted to make it clear at the onset this was not a public hearing. He would allow some comments but they would be restricted to whether or not a public hearing should be held on this matter.

Davene Dadiego, manager of the Dry Mills Store on Route 26, said, "I am angered that one person is trying to take away my right, to smoke." Ms. Dadiego stated that there should not be a public hearing on this matter.

Ms. Dadiego explained that the store does "card" young people who come in to purchase cigarettes and that the store had passed the test given by the State. I do my part when I go to work, she said.

Mike Berrard, a resident of Gray, asked if there was a particular incident that brought this subject up. He wondered how it had come about, had a minor/store broken the cigarette sales law? Mr. Sanborn said that some members of the community had asked him to put this on the agenda.

Stephen Amergian, owner of Dry Mills Store, asked if the Council was voting on this tonight. Mr. Amergian said that he had heard Mr. Sanborn on the news and it came across that his store was selling to minors. He explained that his store was subject to annual inspections by the State to ensure that sales were not being made to minors. "If the Council passes this, you will put me out of business. Kids are always going to come up with ways to get cigarettes," he said. I asked my customers if they would continue to use my store if they can not purchase cigarettes here. People will go over to Windham to get gas, food and cigarettes. In effect, you are penalizing everyone in this town. I like this town, but I will not be able to keep my business here, he said.

Dan Gilliland, 21 Lattimer Road, Gray, a member of the Economic Development Committee and small business owner, addressed the Council next. He felt that Gray's business climate would be going backward that this would create a negative image for the Town of Gray. "We would not be sending the message that Gray is a business-oriented community. We have worked hard on a comprehensive plan, and on zoning. The message being sent is that the Council is failing their business community. We had to have a balance. We need to find another way to protect the children," Mr. Gilliland said.

Cal Cutter, resident of Gray, felt that this was a correct gesture, however he questioned the legality of it. Mr. Cutter wondered whether this would be burdensome and discriminatory and suggested that a State representative do something at the state level regarding the sale of cigarettes to minors.

Julie McCale, Yarmouth Road resident, informed the Council that she was appalled that they were asking that a legal substance be banned. "What is next - alcohol, pesticides or hardware store item that can be potentially dangerous to me?" she asked. You need to continue in your efforts to enforce the laws as they pertain to minors. I do not want to live in town that bans cigarette sales, she said.

Chair Sanborn reminded the public and the Council members that due to statutes that must be followed they had to adhere to the issue at hand. This is not a public hearing and can not be conducted as one without tobacco companies having been given notice of a public hearing.

Councilor Monroe said that she understood Mr. Sanborn's intent but felt that she had to also look at people's rights. She suggested that our representatives take some of this energy over the concern of sales of tobacco products to minors. Councilor Monroe said that she was not comfortable with the message that this would send to businesses. She would not support a public hearing on the matter.

Councilor Goosetrey commented that he was against any ordinance prohibiting the sale of tobacco. He would not support taking individual rights away from people.

Councilor Norton explained that he seconded the motion so that he could hear both viewpoints on this. There are two issues here – number one is image and number two is legal.

Councilor Cook commented that the Chair was thinking of the children in the Town. Mr. Cook said that he did not want to see the Council responsible for this decision. He wanted a public hearing and lent his support to Councilor Sanborn.

Chair Sanborn explained that if the Council allowed this to proceed, they could receive comments from the public. Full input would be allowed at a public hearing and the public would have a right to speak on the issue. The public should have a right to speak on it, he said.

Cathleen Manchester, another Gray resident, ~~rallied~~ spoke to Mr. Sanborn's side. She encouraged a public hearing because children are buying or stealing cigarettes. This leads to health problems and problems in our schools. A public hearing would be of educational value, and she felt it was important to hold one.

Donald Whitney, 64 West Gray Road, also supported a public hearing. He said, "If our grandparents had done this we wouldn't be having this discussion today."

The Gray Town Council voted 3-2, to reject the motion. (Cook and Sanborn in favor, Monroe, Norton and Goosetrey opposed)

Order 204: Council discussion and action concerning Councilor Cook regarding Town Council Rule 602.23, which states:

Council Voting:

Every member present when a question is put shall give his/her vote unless the Council for special reasons, shall excuse him/her. Applications to be so excused must be made before the Council is divided, or before the calling of the yea's and nays and decided without debate.

Councilor Monroe made the motion that Councilor Cook abides by Rule 602.23 and that action be taken to have Mr. Cook step down.

Councilor Goosetrey stated that he did not understand the motion and asked for further explanation.

The motion failed to receive a second and the motion died.

Sixth order of Business

Tabled Orders Council review and action on acceptance of "Cobb's Crossing" as a town way. Submitted by Town Manager.

Council Cook made the motion to review and accept the action on "Cobb's Crossing," as a town way. Mr. Jankowski noted that he had received memos from the Public Works Director and Town Engineer and the Councilors may need time to go over them.

Mr. Cook withdrew his motion. The order is tabled until the next meeting.

Standing Orders:

Order 010: Council approval of Pole Permits for Central Maine Power and Pine Tree Telephone Company.

None

Order 011: Public discussion on Non-Agenda Items

Mary Bosse, a Gray resident, commented that the holiday decorations were beautiful and thanked all those responsible for doing such a nice job in the center of town.

Brad Fogg, 21 Shaker Road in Gray, reminded the Council of the next Cable Committee meeting.

Order 012; Council approval of Concealed Firearms Permits as request by the Maine State Police Licensing Division.

Councilor Monroe made the motion to approve the Concealed Firearms Permits as requested by the Maine State Police. Councilor Norton seconded the motion. The Council voted unanimously to approve.

Councilor Cook made the motion to recess for ten-minutes at 8:10 p.m. Councilor Norton seconded and the Council vote was unanimous.

Order 205: Executive session regarding personnel issue.

It was moved by Councilor Sanborn, seconded by Councilor Monroe and unanimously voted to go into Executive Session for Order 205, at 8:20 p.m.

It was moved by Councilor Sanborn, seconded by Councilor Norton and unanimously voted to go out of Executive Session at 9:10 p.m.

Seventh Order or Business
Adjournment

It was moved by Goosetrey and seconded by Cook to adjourn the meeting of the Town Council at 9:10p.m. The Council vote was unanimous.

Respectfully Submitted,
Janmarie A. Bourret
Janmarie A. Bourret
Recorder

District Manager
Customer Service and Sales
District of Maine

12/1 | Agenda

UNITED STATES POSTAL SERVICE

November 16, 1998

Peter Jankowski
Town Manager
PO Box 258
Gray ME 04039-0258

Dear Mr. Jankowski:

The Postal Service has determined that the present postal facility located on Route 26 is inadequate to serve Gray's future postal needs. We need to expand operations to enhance both the quality of postal services we provide to your community and our employees' working conditions. After consulting with Postmaster Nancy Clark, we find that we need, ideally, approximately 5,000 square feet of net interior space.

To meet these new requirements, our first consideration is to expand the existing postal facility. If this is not feasible, we will then consider relocating to another building or construct a new building on a site that is, ideally, approximately 77,000 square feet. In the event another building or site must be identified, every effort will be made to maintain it within the downtown business area of Gray.

The Postal Service wishes to work in partnership with your community. Therefore, we ask your assistance and cooperation in having this project scheduled for the next available town meeting so that the general public may express its opinions and offer constructive comments on the project. Please address any questions, suggestions, or concerns to:

Terry L. Brooks
Real Estate Specialist
P.O. Box 800
Winthrop ME 04364-0800

Thank you for your participation in this process and we look forward to continue working with you and your staff as this project develops.

Sincerely yours,

Barbara A. Patterson

Put on Agenda

Barbara A. Patterson
District Manager, Customer Service and Sales
District of Maine

380 Riverside Street
Portland ME 04103-7000
Phone: 207-828-8529
Fax: 207-828-8447
