# Supplementary material for: Tobacco industry and public health responses to state and local efforts to end tobacco sales from 1969-2020
Source: PLoS One. 2020 May 22;15(5):e0233417. doi: 10.1371/journal.pone.0233417 (PMC7244130; doi:10.1371/journal.pone.0233417)
Supplement: S3 Data — (PDF) [file pone.0233417.s003.pdf]

TOWN OF WESTMINSTER

11 South Street
WESTMINSTER, MASSACHUSETTS 01473
(978) 874-7409 • Fax (978) 874-7460

BOARD OF HEALTH

MINUTES FOR APRIL 2, 2014 BOH. MEETING

Board of Health Members in Attendance: Andrea Crete Chairman, Peter Munro, Edward Simoncini; Health Agent Wibby Swedberg; Joyce Lucander, Administrative Assistant; Tobacco Alliance Joan Hamlett; Jerry Powers, Westminster resident; Feliatas Fandreyer, Student Nurse.

4:34 p.m. Andrea announced that the meeting was open and was being recorded by Joyce.

4:34 p.m. The Board did not sign the minutes from March 19th .Ed had looked them over and wanted a few changes made. Peter made a motion to table the minutes until next meeting. The Board all agreed.

4:43 p.m. Vouchers were approved and signed: for Wibby gas mileage for February & March, Wibby for the purchase of a tape recorder & batteries to record meetings, Rita for March gas mileage and for a battery purchased, Bailey's for food inspection supplies.

4:46 p.m. Agents Update:

1. Wibby will be meeting to discuss the Septic Loan Program with Jim Starbard from RCAP, Melody Gallant, Karen Murphy & Julie Costello on Thursday. They will discuss using RCAP to administer the program or doing it in-house.

2. Waste Management/RCI has withdrawn their application to the Department of Environmental Protection (DEP) for extending the Special Permit Demonstration Project at the landfill. DEP has promulgated new Solid Waste Regulations that only require notification for the disposal of Asbestos, Waste Water sludge, & Bio or Medical waste categories. DEP and the Board of Health will have 45 days for input on accepting those solid wastes. Worcester DEP has hired an engineer to review the applications.

Annual Nominations of Animal Inspectors for the town are due. Peter made a motion to approve the inspectors for the year. Ed seconded the motion. All were in favor. Peter would like to know where the vehicle for the animal control officer is.

Wibby spoke with Jonathan from Wyman Auto. They will be having the water tested for VOC's and plan to have the tight tank in before the winter.

Board members concerns: None at the moment.

Manganese in Drinking Water fact sheet from Mass DEP was given to the Board. There have been new findings about health concerns of manganese in drinking water especially affecting infants whose formula is prepared with water with high levels. The EPA has issued new requirements for testing manganese in public water supplies. Our local well regulations require EPA standards for drinking water be met by filtration systems if necessary.

Discussed requirements for Tight Tank approval. Septic plans with Tight Tanks are no longer submitted to DEP for approval. Local Boards of Health are now the sole approving authority. Wibby asked the Board for input on a Tight Tank Guidance document she had written for system designers. The Board discussed Guidance for Septic Plans with Tight Tanks. The Board decided that if Tight Tanks could not be outside the 100 foot buffer zone from wetlands that a variance to local regulation 245-14 Setback Requirements, would need to be requested.

There has been some confusion amongst engineers/septic system designers in regards to the wording of 245-14 Setback Requirements. For clarification Ed made a motion to section 245-14 in the Board of Health Regulations to delete the word "new" from the first sentence. The sentence will be changed from **No new**

sewage disposal system shall be installed… to "No sewage disposal system shall be installed within 100 feet of any watercourse, stream, brook, river, pond or wetlands." Peter seconded the motion. All were in favor. Wibby will amend the Board of Health Regulations Chapter 245 and date today.

5:07 p.m. Joan Hamlett, Tobacco Alliance, changes to regulations to consider. Joan went over with the Board all the products that get the younger generation hooked on tobacco including blunt wraps and flavored wraps. The recommended changes to our local regulations address these new products. Joan discussed the pros and cons of raising the age of buying tobacco in town to 21 years old. The Department of Public Health is not currently recommending taking that step. Joan went over Westminster's past and present history on inspections and compliance checks for selling to underage teens. Westminster has had an excellent record. Andrea would like to see the past five years of the history. One of the changes the Board was asked to consider was a ban on selling tobacco products in Health Care Institutions which includes pharmacies. Surrounding cities and towns have instituted that ban. The Board felt it would be fairer to ban all sales of tobacco in the town if that is legal. The Board asked Joan to come to the next meeting with D. J. Wilson, Mass Municipal Attorney, to discuss changes for the town's tobacco regulations. Wibby asked Joan for no smoking signage for the Farmers Market for this year.

6:10 p.m. Peter made a motion to adjourn. Ed seconded the motion. All were in favor.

Andrea Crete	Peter Munro	Edward Simoncini
Andrea Crete, Chairman	Peter Munro	Edward Simoncini

Minutes were signed by Board members on 4/16/2014 Original signatures on file in BOH office.

Attachments:
Mass DEP Fact Sheet Manganese in Drinking Water: Typical questions and answers for consumers
Manganese monitoring
Sample regulations restricting the sale of tobacco products and nicotine delivery products
Tobacco 21 DPH Talking Points Webinar
Tobacco21 Massachusetts Experience
Tobacco 21 Pro arguments from Pediatrician.

TOWN OF WESTMINSTER

11 South Street
WESTMINSTER, MASSACHUSETTS 01473
(978) 874-7409 • Fax (978) 874-7460

BOARD OF HEALTH

MINUTES FOR APRIL 16, 2014 BOH. MEETING

Board of Health Members in Attendance: Andrea Crete Chairman, Peter Munro, Edward Simoncini; Administrative Assistant Joyce Lucander; Jason Dubois Bertin Engineering; Chris MacKenzie, Whitman & Bingham Engineering; DJ Wilson, Mass Municipal Association; Joan Hamlett, Tobacco Alliance.

4:30 p.m. Andrea announced that the meeting was open and was being recorded by Joyce.

4:30 p.m. The Board approved the minutes and signed them for March 19th and April 2nd.

4:35 p.m. Chris MacKenzie, Whitman & Bingham Engineering, updated the Board about the Sargent Road apartments sewer extension. The permit application and design has been signed by the Sewer Commissioners. The documents will be submitted to DEP hopefully next week. It should take them about three months; then Mass DOT will review them. They will not take as long. The Conservation Commission will be last to look them over. The Board will hold a slot for Chris on the agenda for the second meeting in May for another update. If the documents were submitted to DEP and things are moving along then Chris will not need to give an update.

4:40 p.m. Jason Dubois, Bertin Engineering, 180 Minott Road local upgrade request for leaching area offset from well 95 feet instead of 100 feet. The Board reviewed the septic plan. Peter made a motion to approve with Wibby's review of the plan. Ed seconded the motion. All were in favor.

5:05 p.m. Joan Hamlett, Tobacco Alliance, & DJ Wilson, Mass Municipal Association, discussed updating the town tobacco regulations The Board discussed CVS pharmacy's new policy of not selling products at all and talked about not selling any tobacco products in town sometime in the future. Joan advised the Board to speak with Town Counsel. DJ suggested handing out surveys to people in town and get opinions to see how many would support a dry town for the sale of tobacco. Andrea talked about living in a healthy community. The Board will think about those changes. They asked Joan & DJ to draft a letter. Joan discussed smoke free housing that a lot of landlords are now doing. A seminar May 20th will be held. She will send the information to Wibby. Joan will work on a new draft with the changes that were discussed highlighted that include prohibiting the sale of blunt wraps and flavored wraps. She will send the draft to Wibby to give to the Board. She will have this draft ready for the next meeting.

6:05 p.m. Peter made a motion to adjourn. Ed seconded. All were in favor.

Andrea Crete	Peter Munro	Edward Simoncini
Andrea Crete, Chairman	Peter Munro	Edward Simoncini

Minutes were signed by Board members on 5/21/2014 Original signatures on file in BOH office.

Attachments:
180 Minott Road septic design

TOWN OF WESTMINSTER

11 South Street
WESTMINSTER, MASSACHUSETTS 01473
(978) 874-7409 • Fax (978) 874-7460
BOARD OF HEALTH

TOWN OF WESTMINSTER

11 South Street
WESTMINSTER, MASSACHUSETTS 01473
(978) 874-7409 • Fax (978) 874-7460

BOARD OF HEALTH

MINUTES FOR MAY 21, 2014 BOH. MEETING

Board of Health Members in Attendance: Andrea Crete Chairman, Peter Munro, Edward Simoncini; Health Agent Wibby Swedberg; Administrative Assistant Joyce Lucander; Chris Mossman, Trowbridge Engineering; Property owner 122 Ellis Road, Gordon Martin; Chris MacKenzie, Whitman & Bingham Engineering.

4:33 p.m. Andrea announced that the meeting was open and was being recorded by Joyce.

4:33 p.m. The Board approved the minutes and signed them for April 16th and May 7th.

4:35 p.m. Agent Update:

1. Wibby did an inspection today at the Dollar General. They will be opening up on Sunday. She will reinspect on Friday as the temperatures in the freezers were not up to the required temperature.

4:36 p.m. Trowbridge Engineering, Chris Mossman variance request for Gordon Martin 122 Ellis Road.

1. 310 CMR 15.212 (1)a Leaching facility setback from a well, required 100 feet, proposed 88 feet.
2. 310 CMR 15.212 (1)a Leaching facility setback from a property line 100 feet required, proposed 7+ feet.

Chris gave the history of this home. The property was taken by the town for back taxes. The home owner put it up for sale and a developer bought the home so the owner paid the back taxes to redeem it. Ed voiced concern with granting local upgrade approvals/variances when the plan is to tear down the existing 3 bedroom home and rebuild. He feels it should be looked at as "New Construction." He shared that in Title 5 Regulations there were 2 sections that he feels pertains to this. The definition for New Construction in 310 CMR 15.002 discusses an exception for a house that is totally or partially destroyed or demolished. The words "that has been" are used, but in this case it hasn't been demolished yet. Also in New Construction an approval of a new well closer than 100 feet to the leaching area would not be allowed. Chris explained that the existing well is a dug well in the front of the home. The plan is to drill a new well 88 feet from the leaching area. The foot print of the house will be 600 square feet to the former 800 square feet, and the house is going from 3 bedrooms down to 2 bedrooms (7 rooms in total) so the flows with be less. Andrea felt it was much better then what was there now after Chris said a cesspool was in the back and had a failed Title 5. Peter asked about the existing home's condition. It is in extreme disrepair which is why the plan is to demolish it and rebuild. The Board discussed that a two bedroom deed restriction will be required. Ed said if this were new construction on such a tiny lot, a nitrogen treatment plan would be required. Wibby shared with the Board that Gordon came to see her to discuss the purchase and requirements from the Board of Health if he were to buy this lot. He had spoken about his intentions with Chris Mossman about the septic & well and discussed rebuilding the home with the Building Inspector. The Board has approved local upgrades/variances for properties in the past where the plan was to rebuild. Wibby encouraged the Board to be consistent with how they have dealt with such lots in the past and to discuss changing their local regulations to address this for future properties. Peter made a motion to approve the local upgrades requested, with the condition that prior to issuing the Certificate of Compliance we receive a two bedroom deed restriction stamped by the registry of deeds. Andrea asked that an additional condition be a water quality test done after 6 months of the people living in the home. The Board will be considering changes to their local septic regulations. Peter will bring in definitions of "New construction" from the Building Codes to the next meeting.

5:15 p.m. Board members concerns:
Wibby's annual job performance review. Andrea, as Chair, had done the review before the meeting today. Peter & Ed were ok with it. They told Wibby that she is doing a good job.

5:17 p.m. Wibby discussed a complaint call we had received from a neighbor about an abandoned home with an in- ground pool on the property. A fence was falling down all around the yard area. The concern was of area kids or an animal falling into this pool. The police had put Caution tape around the pool and

Wibby put up yellow Caution tape around the entire yard. A Title 5 had been done yesterday. The future buyer will be taking care of the fence & pool. The closing will be on Thursday. The Board & Wibby discussed options of how to handle abandoned homes with pools in the future and deal with banks that own these properties. Peter suggested Rent-a-fence could be a temporary solution.

5:22 p.m. Chris MacKenzie, Whitman & Bingham Engineering, updated the Board about the Sargent Road apartment sewer tie in. Received a phone call from DEP that they no longer review sewer plans, so all set with them. The plan has been submitted to Mass DOT. Chris mentioned crossing over Route 2's on and off ramps on Route 140 hoping for 30-60 days to hear back. Conservation Commission will have a public hearing on a notice of intent on June 12th .Wibby mentioned she had discussed with Josh Hall about McNally's tying in if their septic fails. Peter asked about how long the work will take once approved. The Board asked Chris about the sizing of the line. He will ask Anthony from Whitman & Bingham and let us know. Wibby will plan to attend the meeting with Cons Com.

5:32 p.m. Discuss Draft Tobacco Regulations. Discuss surveying the town on not allowing sale of tobacco. The Board discussed eliminating all tobacco products in town. Andrea shared the idea of having an incentive for the store owners to sell smoking cessation products instead of tobacco products. Wibby will be working with Joan on a draft regulation and what is needed prior to a public hearing. We will need to advertise a public hearing in the paper and on the town website. The Board would like a commitment from Tobacco Control Alliance to represent us in court if needed; to have something in writing from them. Many area cities and towns have banned the sale of tobacco in pharmacies. The Board felt there would be a more even playing field if they banned all sales of tobacco products and nicotine delivery products in town. Every few years the tobacco companies come out with new products to try to entice young people to become hooked on nicotine requiring to Board to keep revising their regulations. A ban would eliminate the need to be changing regulations and would help protect the health of residents in Westminster.

5:45 p.m. Peter made a motion to adjourn. Andrea seconded the motion. All were in favor.

Andrea Crete	Peter Munro	Edward Simoncini
______________________	______________________	______________________
Andrea Crete, Chairman	Peter Munro	Edward Simoncini

Minutes were signed by Board members on 6/18/2014   Original signatures on file in BOH office.

Attachments:
Sample Regulation Restricting the Sale of Tobacco Products
122 Ellis Road septic design

TOWN OF WESTMINSTER

11 South Street
WESTMINSTER, MASSACHUSETTS 01473
(978) 874-7409 • Fax (978) 874-7460

BOARD OF HEALTH

MINUTES FOR JUNE 4, 2014 BOH. MEETING

Board of Health Members in Attendance: Andrea Crete Chairman, Edward Simoncini, Peter Munro; Administrative Assistant Joyce Lucander; Health Agent Elizabeth Swedberg; Chris Mackenzie, Whitman & Bingham Engineering.

4:35 p.m. Andrea announced that the meeting was open and was being recorded by Joyce.

4:35 p.m. The Board approved and signed the vouchers for Wibby's May gas mileage, iPad glass replacement, Rita's May gas mileage, MAHB 2015 Membership dues.
The Board wrote their signatures on the Authorized Signatories form for the Town Accountant.

4:40 p.m. Agents Update:

1. The fence is up at 33 North Common Road. The pool is all enclosed.
2. During Wibby's annual review Andrea suggested letting the Board know of what was going on in town. Wibby updated the Board on a complaint regarding the idling train at the bridge near TRW and the soccer field. Wibby received a copy of the soccer schedule from Parks and Recreation. Michelle Delemarre handles the complaints for DEP on noise & smells. She contacted the Executive of Pan Am Railways. During the games the train will not idle in the area. Ed suggested having a meeting in the fall with the Pam Am people to discuss why the train needs to idle.
3. Wibby met with Doug Halley. He had sent the forms that his Town uses for the septic grant program. Doug would be willing to do the Peer to Peer grant in town again. More people have been coming in looking for help with repairing their septic's that are in failure. Ed asked about the elderly deferment.

4:48 p.m. Board Members Concerns:
Ed dropped off a water sample to Nashoba Analytical Lab. He thought it might be helpful to have a list of what the Town requires for testing wells to put in the envelope so there is no confusion at the lab.

5:00 p.m. Chris Mackenzie, Whitman & Bingham Associates, brought in plan for 37 Willard Road. Local upgrade approval request for 50 foot offset to drilled well rather than the required100 feet. Chris told the Board that Mark Piermarini was working on this plan and could not attend the meeting. After looking over the plan the Board discussed moving the well. They are uncomfortable with only 50 feet setback from the well to the septic leaching area. Since the main reason for the local upgrade approval request was due to the cost of moving the well or the septic system the Board requested 2 to 3 estimates on the cost of installing the septic system. Joyce will look up the depth of surrounding wells to help determine the cost of drilling a new well. This was continued until the June 18th Board meeting at 5:00 p.m.

5:27 p.m. Nowland, 202 Ashburnham State Road replacing dug well with a drilled well. Wibby told the Board that Bob Maki, Conservation Agent, said the new well location is 100 feet from the wetlands so the Board does not need to approve a variance for a well offset

5:28 p.m. Planning Board is requesting input on Dig & Pour LLC/Alan Belanger's application to use the property at the end of Rowtier Drive for industrial use. The Board asked Wibby to speak with Steve Wallace to get clarification on what Alan is already permitted to do and how this application is different

5:40 p.m. Tobacco sale ban. Wibby had spoken with Joan Hamlett Wibby asked the Board to look over the draft Regulation of the Westminster Board of Health Banning the sale of Tobacco products and Nicotine Delivery Products that Wibby handed out to them. Ed made a motion to put a moratorium on issuing tobacco licenses for the next six months while we promulgate new tobacco regulations. Peter seconded the motion. Ed Simoncini, aye, Peter Munro, aye, Andrea Crete, aye. The Board asked Wibby to check with the Tobacco Control Alliance to see if the vote for a moratorium would be valid.

TOWN OF WESTMINSTER
11 South Street
WESTMINSTER, MASSACHUSETTS 01473
(978) 874-7409 • Fax (978) 874-7460
BOARD OF HEALTH

5:58 p.m. Peter made a motion to adjourn. Ed seconded the motion. Meeting adjourned.

Andrea Crete	Peter Munro	Edward Simoncini
Andrea Crete, Chairman	Peter Munro	Edward Simoncini

Minutes were signed by Board members on 7/2/2014 Original signatures on file in BOH office.

Attachments:
37 Willard Road septic plan
2014 Income Limits Summary
Draft Regulations of the Westminster Board of Health Banning the Sale of Tobacco Products and Nicotine Products

TOWN OF WESTMINSTER
11 South Street
WESTMINSTER, MASSACHUSETTS 01473
(978) 874-7409 • Fax (978) 874-7460

BOARD OF HEALTH

MINUTES FOR JUNE 18, 2014 BOH. MEETING

Board of Health Members in Attendance: Andrea Crete Chairman, Edward Simoncini; Health Agent Wibby Swedberg; Administrative Assistant Joyce Lucander; Mark Piermarini, Whitman & Bingham Engineering; DJ Wilson, Mass Municipal Association; Joan Hamlett, Tobacco Control Alliance.

4:35 p.m. Andrea announced that the meeting was open and was being recorded by Joyce.

4:35 p.m. Voucher was approved and signed by the Board to reimburse Cheryl Belanger for perc testing. She paid for 3 lots one of which had already been perced.

4:36 p.m. The Board approved the minutes and signed them for May 21st

4:35 p.m. The Board discussed the Bread of Life addition and parking spaces. Planner Steve Wallace would like comments by the Board. Ed and Andrea were all set with the project as it has town water & sewer.

4:40 p.m. Whitman & Bingham, Mark Piermarini regarding 37 Willard Road continued from June 4th meeting. Mark had made the septic system smaller so it was now 73 feet from the well instead of the previous 50 feet. Mark did not have cost estimates on the septic installation or a new well. This will be continued to the next Board meeting on July 2nd.

Ed shared an idea of having a check list taken from the Title 5 Regulations 310 CMR 15.405 to help the Board in considering local upgrade requests. Ed will work on this so we will have it to put in with the 9A form.

5:04 p.m. DJ Wilson & Joan Hamlett to discuss new tobacco regulations. Ed made a motion to rescind the vote on June 4th that would have put a moratorium on issuing tobacco licenses for the next six months while new tobacco regulations were promulgated. Andrea seconded the motion. Ed Simoncini, aye, Andrea Crete, aye.
Ed made a motion to cap the number of tobacco permits at the current number plus 1, making the cap at 8 tobacco licenses in town. Andrea seconded the motion. Ed Simoncini, aye, Andrea Crete, aye.

DJ went over with the Board & Wibby the procedure for prohibiting all sales of tobacco products and nicotine delivery products in town. The draft regulations were reviewed and revisions suggested. After Karen Murphy and the Selectmen are made aware of the Board's intentions the drafts will be sent to the Town Counsel to weigh in on. DJ will be working on a draft for the Public hearing notice in the paper and will send language for Andrea as Chair to read at the Public Hearing. Andrea had a concern of the cost of a court case if the regulations are appealed. Joan said we will not know until we get there. The regulation may be appealed and it may not. When the Town of Athol prohibited smoking in private clubs the case was appealed and ended up in the State Supreme Court. The Town of Athol had very little legal cost as the attorneys for the Mass Association of Health Boards, Mass Municipal and Boston's Public Health Department all filed briefs and helped out for no cost as it was precedent setting. Joan will meet with store owners to make sure they have received a copy of the draft regulations prior to the public hearing. Joan would be glad to meet with the Selectmen & Karen Murphy for their input and to answer questions about the ban and how this would eliminate the need to be changing regulations and would help protect the health of residents in Westminster. The draft regulations will say the ban goes into effect December 31, 2014. The Board would be willing to extend the permits if need be until March 31st, 2015.

6:15 p.m. Dollar General said they would be seeking a tobacco permit in the near future. It was suggested Wibby send a letter to them explaining the Board is considering new regulations which may affect the license in the future. They will be advised of any developments.

TOWN OF WESTMINSTER

11 South Street
WESTMINSTER, MASSACHUSETTS 01473
(978) 874-7409 • Fax (978) 874-7460

BOARD OF HEALTH

Wibby is working on the documents for the Septic Loan Program. She has a few more to get from the Town of Acton. When the documents are complete they will be been sent to Town Counsel for review.

6:20 p.m. Ed made a motion to adjourn. Andrea seconded the motion. All were in favor.

Andrea Crete		Edward Simoncini
Andrea Crete, Chairman	Peter Munro	Edward Simoncini

Minutes were signed by Board members on 7/2/2014 Original signatures on file in BOH office.

Attachments:
Draft regulations: Prohibiting the Sale of Tobacco in Westminster
Revised Septic plan for 37 Willard Rd.
Title 5 Interpretive Guidance: Consideration of Cost in Determining Maximum Feasible Compliance

TOWN OF WESTMINSTER
11 South Street
WESTMINSTER, MASSACHUSETTS 01473
(978) 874-7409 • Fax (978) 874-7460
BOARD OF HEALTH

MINUTES FOR JULY 2, 2014 BOH. MEETING

Board of Health Members in Attendance: Andrea Crete Chairman, Edward Simoncini, Peter Munro; Health Agent Wibby Swedberg; Administrative Assistant Joyce Lucander; Mark Piermarini, Whitman & Bingham.

4:35 p.m. Andrea announced that the meeting was open.

4:35 p.m. Voucher was approved and signed by the Board for June gas mileage for Wibby, Rita, & Joyce, MHOA 2015 Membership dues were re-signed from 6/4/2014

4:36 p.m. The Board approved the minutes and signed them for June 4th & June 18th.

4:40 p.m. Whitman & Bingham, Mark Piermarini regarding 37 Willard Road continued from June 18th meeting. Mark had brought in 2 cost estimates on the septic installment and new well. Ed made a motion to approve the variance on 37 Willard Road. Peter seconded the motion. All were in favor of the variance of 73 feet from the well.

Agent Update:
1. Pat Glover, former Board of Health member sends her greeting to the Board.
2. We received another check from the Center for Health Care Financing Commonwealth Medicine that processes reimbursement from Health Insurance companies for administering the flu vaccine. This one was for $4,954.60, which raises the total in the Board of Health Public Health Clinic and Emergency Response Revolving Account to $12,644.26.
3. Flu vaccine allotment from the state is the same as last year: 230 doses for 2014/15 season. We have ordered 150 doses from Sanofi Pasteur as well.
4. Per Andrea's request the agents will be doing a monthly activity report of inspections. June's report was given to the Board. Ed asked that a list of permits issued also be included.
5. Wibby is arranging for the Selectmen to attend a Board of Health meeting to discuss prohibiting tobacco sales in town.
6. We have received phone messages from Dollar General regarding applying for a Tobacco Sales Permit. Joan Hamlett from the Tobacco Control Alliance suggested letting them know the Board voted to cap the number of Tobacco sales permits in town and that they were receiving the last one. She also said to let them know the Board is considering some changes in the tobacco regulations that may affect sales in the future. Andrea suggested the Board send a certified letter so we can confirm Dollar General received the information.

Board Member Concerns:
Ed questioned why he had not received information requested at a previous Board meeting regarding the site plan for Alan Belanger at Rowtier Drive. Wibby had spoken with Stephen Wallace the day after the Board meeting, and had emailed the answers to the Board's questions. Wibby has had some email issues. Ed will check to see if he indeed received the email or not. Wibby will check through her sent folder and send the email again.

Ed shared a spreadsheet he had worked on taken from the Title 5 Regulations 310 CMR 15.405 to help the Board in considering local upgrade/variance requests. Ed asked the Board and Wibby to review it and try to establish a check list so we will have it to put in with the 9A form.

Ed voiced concern that some of the Well Companies are not submitting the Well Completion Report within the required 30 days. His step-son's report was submitted 45 days after drilling. Ed asked the Board to consider if they wanted to institute a late fee.

TOWN OF WESTMINSTER

11 South Street
WESTMINSTER, MASSACHUSETTS 01473
(978) 874-7409 • Fax (978) 874-7460

BOARD OF HEALTH

Ed asked the Board to consider charging engineers and septic system designers for errors they make on the septic plans.

5:13 p.m. Cumberland Farms amendment to the license from 32,000 to 40,000 gallons for underground gasoline tank storage. The Selectmen asked the Board for input. The original application was for 40,000 gallons that included 8,000 gallons of storage for diesel gasoline. There will not be diesel gasoline sold so the Selectmen had removed the 8000 gal. Cumberland Farms are asking that the 8000 be added back as the storage tanks would remain the same size. One tank will now be a one compartment tank instead of 2 compartments. The Board had no concerns or comments.

5:15 p.m. Peter made a motion to adjourn. Ed seconded the motion. All were in favor.

Andrea Crete   Peter Munro   Edward Simoncini

Andrea Crete, Chairman   Peter Munro   Edward Simoncini

Minutes were signed by Board members on July 16, 2014   Original signatures on file in BOH office.

Attachments:
Spreadsheet taken from Title 5 Regulations 310 CMR 15.405
Health insurance reimbursement note & check for flu clinics

TOWN OF WESTMINSTER
11 South Street
WESTMINSTER, MASSACHUSETTS 01473
(978) 874-7409 • Fax (978) 874-7460
BOARD OF HEALTH

MINUTES FOR JULY 16, 2014 BOH. MEETING

Board of Health Members in Attendance: Andrea Crete Chairman, Edward Simoncini, Peter Munro; Health Agent Wibby Swedberg; Administrative Assistant Joyce Lucander; Chris Mossman, Trowbridge Engineering; Denis Moquin home owner 12 Lake Drive West.

4:35 p.m. Andrea announced that the meeting was open and was being recorded by Joyce.

4:35 p.m. Wibby told the Board that the Agenda had not been posted for this meeting.

4:36 p.m. The Board approved the minutes and signed them for July 2nd.

4:37 p.m. Agents Update:

1. Wibby has received all the paper work and along with Rita has been inspecting Camp Pineshore & Spartan Basketball camp.
2. 16 Laurie Lane. Rita witnessed a Title V done in June by a local inspector. It was deemed a failure due to leaching pit being in the groundwater. Karen Murphy received a phone call from the homeowner's son saying he was very upset about the way Rita and the inspector determined the failure. Wibby had received a call from another inspector to discuss the findings of the inspection that was done. It would be in the best interest for those selling the home to hire an engineer/soil evaluator to determine the ground water. That would confirm a failure or not. Wibby wanted the Board to be aware of this situation.
3. Wibby had sent to the Board copies of the septic & well permit log sheets that have been issued for 2014 so far. She asked the Board if they were ok with receiving a copy of the permit log rather than retyping in the monthly Activity Sheet. The Board was fine with the permit log sheet that was sent.

4:45 p.m. Ed met with Karen Murphy regarding the Boards intention of prohibiting all sales of tobacco products and nicotine delivery products in town. The Board asked that Karen send the draft regulations to the Town Counsel for review. All 3 selectmen are not available to meet with the Board at either of the August BOH meetings. Heather Billings and Wayne Walker can attend the August 20 meeting. Ed suggested sending an open invitation to John Fairbanks to come to any other Board meeting. The Board asked Wibby to see if Joan would be available to attend their meeting with the Selectmen and bring in the tobacco products and marketing done to entice children so the Selectmen could see why the Board is considering a tobacco sales ban. Peter would like the Selectmen to issue Tobacco Permits in the future if this would fail. Our mission is to protect public health; issuing permits to sell tobacco seems totally opposed to that mission.

Board members concerns: None

5:05 p.m. Discuss fee for errors/variance omissions on septic plan design. The discussion dealt with the issue of errors and revisions needed on septic system plans. The proposal was raised to issue fines or charge for errors to cover any extra time it takes the agents and office staff to deal with errors/revisions. Wibby voiced concern that this might jeopardize the good relationship she has with engineers/system designers. Most errors are minor and do not take a lot of time to jot down and email or fax to the designer and check off when the revised plan is received. There is already a BOH fee of $50 to resubmit a revised plan when the homeowner has requested changes to the design. The Board thought since that fee was already in place it could be used when the designer makes an error resulting in a revised plan. No decision was made at this meeting. The Board asked Wibby to keep track of how much time it is taking to deal with errors/revisions during plan review. They asked that she note if certain engineers/designers keep making the mistakes of leaving out details that are needed and are not learning what is required by the Westminster BOH. Wibby will keep track of any issues. The Board will address this at another meeting.

TOWN OF WESTMINSTER

11 South Street
WESTMINSTER, MASSACHUSETTS 01473
(978) 874-7409 • Fax (978) 874-7460

BOARD OF HEALTH

5:30 p.m. The Board discussed Camp Pineshore's request for a variance from the Food Code sink requirements. The Board did not vote tonight on the variance due to agenda not being posted.

5:36 p.m. Chris Mossman Trowbridge Engineering brought in a septic plan for 10 Lake Drive West. Abutter Denis Moquin was present. Variances were requested for a tight tank to replace the failed septic system:
310CMR 15.212(1) Tight tank setback from property line required 10, proposed 2+
310CMR 15.227(5) Tight tank setback from well line (House No. 1) required 50, proposed 44+
310CMR 15.227(5) Tight tank setback from a well line (House No. 12) required 50, proposed 35+
Chris discussed that this was the best he could do with such a small lot. The proposed plan includes a pump inside the house to send the effluent up to the tight tank. An alarm panel is proposed for inside the home. The abutter was concerned about the tight tank overflowing. He voiced concern over a toilet running in the house and the alarm going off with the owners away. The Board discussed options and finally agreed on Wibby's suggestion of an alarm and light on the outside of the house that neighbors could see. The abutter felt that would satisfy his concerns. The Board asked for a water quality test on the wells under 50 feet from the tight tank for a base line. A deed restriction for 2 bedrooms will be required before a certificate of compliance is issued. A plaque will be placed under the outdoor alarm that lists the pumper to call. A contract with a pumper/septage hauler will be required. Chris will revise the plan and bring it to the next BOH meeting.

5:57 Septic Loan Program. Doug Haley will be dropping off a thumb drive with Acton's betterment documents this week. Wibby has been working on a Peer to Peer Grant application that would pay for Doug Haley's time in helping us implement the Septic Loan Program. One of the grant requirements is for the Selectmen to vote in support which they have done. Andrea signed the Peer to Peer Grant application.

5:59 Draft Well Policy Ed was working on will be discussed at another meeting.

6:00 p.m. Peter made a motion to adjourn, Ed seconded the motion. All were in favor.

Andrea Crete	Peter Munro	Edward Simoncini
______________________	______________________	______________________
Andrea Crete, Chairman	Peter Munro	Edward Simoncini

Minutes were signed by Board members on 8/20/2014 Original signatures on file in BOH office.

Attachments:
Westminster Board of Health Draft Well Policy
Pineshore Bible Camp variance letter for variance from Food Code sink requirements in kitchen
10 Lake Drive West septic plan
Plan of lots upper Partridge Reservoir
Septic & Well 2014 issued permits
Title 5 Interpretive Guidance: Consideration of Cost

TOWN OF WESTMINSTER

11 South Street
WESTMINSTER, MASSACHUSETTS 01473
(978) 874-7409 • Fax (978) 874-7460
BOARD OF HEALTH

MINUTES FOR AUGUST 6, 2014 BOH. MEETING

Board of Health Members in Attendance: Andrea Crete Chairman, Edward Simoncini, Peter Munro; Health Agent Wibby Swedberg; Administrative Assistant Joyce Lucander; Chris Mossman, Trowbridge Engineering; Mark Piermarini, Whitman & Bingham Engineering.

4:35 p.m. Andrea announced that the meeting was open and was being recorded by Joyce.

4:35 p.m. The Board approved and signed the vouchers for Wibby's July gas mileage, reimbursement for training Foodborne Illness Investigations Training, Rita's July gas mileage, The Gardner News, Premier Laboratory, & Ben Meadows.

Wibby had taken a water sample test from runoff in the driveway at 185 South Ashburnham Road and a nearby stream that flows by across the street from the property. A copy of the results was handed out to the Board. The E. coli bacteria level was too numerous to count (TNTC). The Board discussed an enforcement letter to be sent to the homeowner about violations that need to be addressed. Andrea will work on the letter and email a draft to Wibby.

4:55 p.m. Mark Piermarini Whitman & Bingham Associate, brought in a plan for 1 Shady Ave. Local upgrade requests for the Board to approve:
310 CMR 15.212(1)(a) Reduction to high ground water elevation required 4 feet, proposed 3 feet.
310 CMR 15.102(2) required 2 deep holes, proposed 1 deep hole.
After discussion with the Board Mark will add to the plans, when the bottom of the bed is excavated a deep hole will be dug to confirm the seasonal high water table. Peter made a motion to approve the local upgrades with the addition on the plans. Ed seconded the motion. The Board was all in favor.

5:04 p.m. Chris Mossman Trowbridge Engineering brought in a revised plan for 10 Lake Drive West. Home owner is Rita Smith. Tight tank to replace failed septic system. Discussion was continued from last meeting. On the revised plan an outside visual and audio alarm was added to the side of the house along with a placard listing the septage hauler to call if the alarm goes off. Local upgrades requested:
310 CMR 15.212(1) Tight tank setback from property line, required 10 feet proposed 2 feet
310 CMR 15.227(5) Tight tank setback from a well line (house # 1) required 50 feet proposed 44 feet.
310 CMR 15.227(5) Tight tank setback from well line (house # 12) required 50 feet proposed 35 feet.
After discussion with the Board Chris will let the home owner know that she will need to have a contract in place with name & phone number of a septic hauler for the placard on the house. This needs to be kept up to date. The Board wanted this recorded in the deed for the property. Ed made a motion to approve the local upgrades. Peter seconded the motion. The Board was all in favor.

5:07 p.m. Chris Mossman Trowbridge Engineering brought in a plan for 14 Linda Drive. Home owner is Gene LeBlanc. This is classes as new construction because there is an increase in flows from the old camp that will be torn down. Chris asked the Board for a variance from local regulation 245-5 use of a retaining wall. Chris described the lot to the Board saying the owner can maintain the system without going on the neighboring property. Peter made the motion to approve the variance. Ed seconded the motion. The Board was all in favor of the variance.

5:14 p.m. Agents Update:
Discuss application fee for septic loan program. Wibby has sent an email to Kopelman & Paige to learn what would be the average legal fee to record the Betterment Notification with the Registry of Deeds. The Board will look at that as a base for the application fee. The discussion included part of the application fee could be refundable for legal fees if they were not spent.

TOWN OF WESTMINSTER

11 South Street
WESTMINSTER, MASSACHUSETTS 01473
(978) 874-7409 • Fax (978) 874-7460
BOARD OF HEALTH

5:18 p.m. Ed made a motion to approve Camp Pineshore's variance request from the Food Code sink requirements in the camp kitchen facility for this year. Peter seconded the motion. The Board was all in favor of the variance for this year.

5:20 p.m. Board Members Concerns:
The Board wanted to make sure that D. J. Wilson, Joan Hamlett & Cheryl Sbarra will be at the next meeting.

Wibby issued the Board a copy of the revised Board & Committee handbook and asked that the Board sign the paper work that they have received it so it could be turned over to the Clerk's office.

5:44 p.m. Ed looked in the Code of the Town of Westminster book and noticed that there are two chapters referring to wells. Chapter 254 Water Supplies; and Chapter 255 Wells, Private and Semi-Public. Some of the requirements in the 2 chapters are not consistent. One requires a well to have a minimum yield of 3 gallons per minute (gpm) while the other chapter's minimum yield is 5 gpm. The Board asked that Joyce investigate to see if one of the chapters is outdated. Ed would like to discuss at a September meeting the procedure of well permits 313.CMR 3, filing completion reports that well drillers need to follow. Meter reading at the site of the wells was discussed. We have used water meters from DPW for flow testing, but had a lot of issues with the meters stopping during the 4 hour test. Andrea suggested having the well drilling companys provide a meter.

6:15 p.m. Andrea made a motion to adjourn. Peter seconded the motion. All were in favor.

Andrea Crete	Peter Munro	Edward Simoncini
Andrea Crete, Chairman	Peter Munro	Edward Simoncini

Minutes were signed by Board members on 8/20/2014 Original signatures on file in BOH office.

Attachments:
Water test results from 185 South Ashburnham Rd and stream nearby.
Septic plans for 1 Shady Ave.
Revised septic plans for 10 Lake Drive West.
Septic plans for 14 Linda Drive.
Camp Pineshore variance request letter.
Board and Committee Handbook

TOWN OF WESTMINSTER
11 South Street
WESTMINSTER, MASSACHUSETTS 01473
(978) 874-7409 • Fax (978) 874-7460
BOARD OF HEALTH

TOWN OF WESTMINSTER
11 South Street
WESTMINSTER, MASSACHUSETTS 01473
(978) 874-7409 • Fax (978) 874-7460
BOARD OF HEALTH

MINUTES FOR AUGUST 20, 2014 BOH. MEETING

Board of Health Members in Attendance: Andrea Crete Chairman, Edward Simoncini, Peter Munro; Health Agent Wibby Swedberg; Assistant Health Agent Rita McConville; D.J. Wilson, Mass Municipal Assoc; Joan Hamlet, Tobacco Control Alliance; Cheryl Sbarra, Mass Association of Health Boards; Wayne Walker, and Heather Billings, Selectmen.

4:32 p.m. Andrea announced that the meeting was open and was being recorded by Rita.

4:35 pm The Board approved and signed the minutes for July 16th & August 6th.

4:45 pm Solar Panel Array at 339 South Ashburnham Rd was discussed. The Board had no issues with the panels. End of life-disposal of the panels and pesticide use was discussed. Wibby will discuss with Steve Wallace about run-off concerns.

5:00 pm Septic Loan program, Wibby outlined and highlighted changes on the draft. Wibby confirmed that 14% is correct for late payment interest according to Treasurer Melody Gallant. Elderly Deferral payment plan was discussed. Other fees such as Attorney fees and recording fees need to be discussed and set for the Betterment. Ed suggested including legal fees in the application fee that would be refunded if loan is rejected. Board voted to decrease the maximum income limit to $100,000. All were in favor.
Ed suggested we make available the tri-folded pamphlet from the state with information about the loan program.

5:15 pm Wibby discussed 185 South Ashburnham Rd. Wibby sent a letter ordering them to cease and disease using plumbing in home, stop drainage of contaminated water into the road, and to hire an Engineer within 48 hours. They hired Kent Oldfield, as Engineer. A date was set for site evaluation for Friday, August 22, 2014.

5; 30 pm Wells. It has come to the board's attention that GAP Mountain Drilling GPS data format was incorrect showing the location of Westminster wells to be in Marlboro. This was discussed with Gap Mountain almost 2 months ago but we are still receiving Well Completion Reports with incorrect well locations. Ed suggested they be invited to the next Board meeting; all voted in favor. A letter will be sent by Wibby explaining the Board's concerns and asking them to come to the next Board of Health meeting

The Board wanted to pass a new policy for Well drillers specifying that building permit applications will not be signed until the BOH receives water test showing Total Coliform Bacteria absent and Chlorine not detected as well as a completed Well Completion Report from the well driller. Notices are being distributed to contractors and well drillers.

5:45 pm Joan Hamlet discussed tobacco company tactics to disguise tobacco to appear similar to candy and use flavors, etc. that would appeal to youth. She handed out colorful info used to educate retail merchants about tobacco sales.
Regulations were discussed that have already been passed by area cities and towns; such as banning blunt wraps and single cigar sales, and E-cigarettes. Ed expressed concern of this constant change in policy called "whack a mole" and how BOH must keep up with tobacco antics, and how these constant changes affect local merchants. DJ and Cheryl discussed with the Board and Selectmen present the proposed complete ban on tobacco sales in Westminster
The next steps to take were discussed: reviewed the proposed draft, discussed bringing in key players, invite local merchants and get public opinion. Discussion tabled until October 1st meeting with Tobacco Control Alliance.

TOWN OF WESTMINSTER
11 South Street
WESTMINSTER, MASSACHUSETTS 01473
(978) 874-7409 • Fax (978) 874-7460
BOARD OF HEALTH

6:30 p.m. Andrea made a motion to adjourn, Ed seconded the motion. All were in favor.

Andrea Crete	Peter Munro	Edward Simoncini
Andrea Crete, Chairman	Peter Munro	Edward Simoncini

Minutes were signed by Board members on 9/17/2014 Original signatures on file in BOH office.

Attachments:
New policy regarding signing Building Permit Applications
Town of Westminster Community Septic loan program application package
Draft version #2 Regulation of the Westminster Board of Health Prohibiting the sale of Tobacco Products
Copy of July 3, 2014 letter to Westminster Liquors Re: Suspension to sell tobacco products for three days
Information to educate tactics to disguise tobacco and sales to minors

TOWN OF WESTMINSTER
11 South Street
WESTMINSTER, MASSACHUSETTS 01473
(978) 874-7409 • Fax (978) 874-7460
BOARD OF HEALTH

MINUTES FOR SEPTEMBER 3, 2014 BOH. MEETING

Board of Health Members in Attendance: Andrea Crete Chairman, Edward Simoncini, Peter Munro; Health Agent Wibby Swedberg; Administrative Assistant Joyce Lucander; Linda Wiest, Master Plan Committee Member.

4:30 p.m. Andrea announced that the meeting was open and was being recorded by Joyce

4:35 p.m. The Board approved and signed vouchers for Wibby gas mileage for August, office supplies, MHOA meeting for 9/18 on Toxins, Rita gas mileage for August, MPHN Annual Assessment.

4:35 p.m. Agents Update:

1. Wibby told the Board the Rita would be on vacation.
2. Received a call from Cumberland Farms in Framingham asking about the tobacco ban she had heard that the Town was considering Wibby told her that nothing was definite yet. They will be notified as to when a public hearing and comment period will be held.
3. Gap Mountain will be at the next meeting to discuss the Well Completion Reports. He has ordered new equipment that will give the correct GPS location of the wells.
4. Wibby gave the Board a copy of the first applicant's financial records for the Septic Loan Program. Ed suggested that Wibby put together a 1 page format with the needed information on it so as to keep the information more confidential.

4:50 p.m. Linda Wiest from the Master Plan Committee discussed the Master Plan with the Board. She asked them to read it and if they could send 1 recommendation to the planner. The Board thanked Linda for coming to the meeting.

5:10 p.m. Agents Update: Continue discussion of septic loan program.
The Board feels they do not need to see all the financial information included with the application. The Board members were all in agreement that Wibby can verify that the applicant meets the requirements. The fee for the application will be $50.00, nonrefundable. The attorney's fees for reviewing and recording the deed could be paid for out of the loan.

5:17 p.m. Discuss Tobacco Ban Draft Regulations. The Board will wait to hear back from D.J. Wilson from Mass Municipal Association & Cheryl Sbarma from Mass Association of Health Boards with their input. The Board discussed possible dates in November to hold the Public Hearing. Wibby will contact Joan Hamlett to have her focus on garnering support for the ban.

5:33 p.m. Discuss Well Policy. The State licenses well drilling companies and has a means in place to fine them. The Board currently has 2 very similar regulations in place Chapter 254 Water supplies and Chapter 255 Wells, Private and Semipublic. The Board asked that we discuss the discrepancies between the regulations at the next meeting.

5:36 p.m. Andrea made a motion to adjourn, Ed seconded the motion. All were in favor

Andrea Crete   Peter Munro   Edward Simoncini

____________________   ____________________   ____________________

Andrea Crete, Chairman   Peter Munro   Edward Simoncini

Minutes were signed by Board members on 9/17/2014   Original signatures on file in BOH office.

Attachments:

Chapter 254 Water Supplies

Chapter 255 Wells, Private and Semipublic

Prepared by the Master Plan Committee (Updated February 2014)

TOWN OF WESTMINSTER
11 South Street
WESTMINSTER, MASSACHUSETTS 01473
(978) 874-7409 • Fax (978) 874-7460
BOARD OF HEALTH

MINUTES FOR SEPTEMBER 17, 2014 BOH. MEETING

Board of Health Members in Attendance: Andrea Crete Chairman, Edward Simoncini, Peter Munro; Health Agent Wibby Swedberg; Administrative Assistant Joyce Lucander; Chris Mossman, Trowbridge Engineering; Ryan Wragg, Gap Mountain.

4:40 p.m. Andrea announced that the meeting was open and was being recorded by Joyce

4:40 p.m. The Board approved and signed the minutes for August 20th & September 3rd. Approved and signed voucher for Wibby for postage mailing to Mass Health.

4:47 p.m. Agents Update:

1. 185 South Ashburnham Road. Wibby had done soil testing with Kent Oldfield. Ledge was within 2 to 4 feet from the surface at the top of the hill; will have to put in a tight tank. Ed asked that they get going on it.

4:57 p.m. 100 Town Farm Road septic plan review was postponed until the next meeting.

The Board discussed how the local requirement of 125% of a Title 5 system would be done in a Presby System. In the past the engineers and designers have just added 25 % to the sand bed. The discussion included if 25 % more Presby pipe should be added as well.

Wibby had asked Chris Mossman to attend to give a system designer's perspective on what the Board will require in the future for approval of Local Upgrades and Variances. When asked to consider cost as a reason to issue the Local Upgrade or Variance the Board discussed requiring 2 bids on the installation of the system. Chris and Wibby voiced that is can be difficult to obtain bids from installers if the installation is not imminent. Chris felt that an alternative would be for the engineer to show a sketch of the system without any variances. He said the Board is knowledgeable enough to compare the 2 designs and evaluate the extra time and money required for extra tree cutting, extra sand fill, etc.

5:30 p.m. Ryan Wragg Gap Mountain Drilling was asked to come to the meeting to discuss the GIS system that he uses to find the location of wells that have been done in the town. The well drilling reports have been coming with locations in Marlborough. Ed discussed that we need the reports within thirty days as required by the State. The Board asked Ryan to correct the Well Completion reports to show the correct coordinates. Ryan was asked by the Board how he determines the gallons per minute flow and the recovery time in the wells. Wibby asked to observe a well being drilled along with the determination of the water flow per minute test. Ryan will let her know of future wells being drilled in town. The Board will be looking over the corrected Well Completion reports that he submits with the correct locations. They thanked Ryan for coming to the meeting.

6:00 p.m. The summary sheet for the septic loan program was discussed. The Board and Wibby felt it looked great. This will be a living document that can be added to and put in the permanent file.

6:03 p.m. Review discrepancies between BOH local regulations Chapter 254 Water Supplies and Chapter 255 Wells Private and Semi-Private. The only major difference is Chapter 254 required 3 gallons per minute (gpm) over 1 hour while Chapter 255 requires 5 gpm over 4 hours. Ed made a motion to remove Chapter 254 from the Board of Health regulations. Peter seconded the motion. All the Board members voted in favor of the motion.

6:06 p.m. Board Members Concerns: None at the moment.

6:07 p.m. Discuss introductory remarks to be read prior to the Hearing on draft Tobacco Regulations that Andrea had written. The Board was very positive about the remarks. Also discussed were the order of

speakers at the meeting and the time allowed per person. November 12th is the date that both DJ Wilson and Cheryl Sbarra can attend.

6:20 p.m. Andrea made a motion to adjourn, Ed seconded the motion. All were in favor

Andrea Crete   Peter Munro   Edward Simoncini

Andrea Crete, Chairman   Peter Munro   Edward Simoncini

Minutes were signed by Board members on 10/1/2014   Original signatures on file in BOH office.

Attachments:
Form 9A 100 Town Farm Road
100 Town Farm Road septic plan
Town of Westminster Septic Loan Program

TOWN OF WESTMINSTER
11 South Street
WESTMINSTER, MASSACHUSETTS 01473
(978) 874-7409 • Fax (978) 874-7460
BOARD OF HEALTH

MINUTES FOR OCTOBER 1, 2014 BOH. MEETING

Board of Health Members in Attendance: Andrea Crete Chairman, Edward Simoncini, Peter Munro; Health Agent Wibby Swedberg; Administrative Assistant Joyce Lucander; Chris Mossman, Trowbridge Engineering; Neil Gorman, David E. Ross Assoc. Inc; D.J. Wilson, & Jenn Robertson, Mass Municipal Association; Joan Hamlet, Tobacco Control Alliance; Cheryl Sbarra, Mass Association of Health Boards.

4:35 p.m. Andrea announced that the meeting was open and was being recorded by Joyce

4:35 p.m. The Board approved and signed the minutes for September 17th. Approved and signed voucher for Wibby & Rita gas mileage for September, Sanofi Pasteur for vaccine.

4:38 p.m. Agents Update:

1. Have not heard back from Gap Mountain about the new well reports with the correct address on the location.
2. Received the vaccine for the flu clinics. They will be held at the Senior Center & the Meeting House School on October 16th and on November 18th at the Fall Town Meeting.

4:42 p.m. Board Members Concerns:
Gap Mountain reports

4:43 p.m. Trowbridge Engineering, Chris Mossman. 98 Narrows Road, Variance request/ local upgrade approval for offset to groundwater 310 CMR 15.212(1)(a), 5 feet required, 4.5 feet proposed.
SDS setback to wetlands: Westminster Board of Health Regulation requires 100 feet, proposed 50 ft.
SDS system size: WBOH requires 560 sq.ft., proposed 448 sq.ft.
Chris discussed the cost saving and that the property had town water. The Board was all in favor of the variances.

4:47 p.m. Ross Associates, Neil Gorman. 100 Town Farm Road, Variance request/ local upgrade approval 310 CMR 15.405(1)(h) Reduction of the required separation between the bottom of the leaching facility and the high groundwater from 4 feet to 3 feet.
Westminster Board of Health Regulation 245-3 125% of Title V design. Presby Enviro-Septic Leaching System linear feet is designed at 100% of the DEP approved requirements. The Board reviewed 2 septic installation bids that the Board had asked for. They discussed the cost savings of removal of trees and more fill that would be needed. The Board was in favor of the variances.

5:05 p.m. Continue with Board Members Concerns. Ed discussed that a letter should be sent to Ryan Wragg from Gap Mountain in regards to the well reports that had the wrong information on the location. It had been two weeks since our meeting with Ryan and we have not heard a word from him or received a complete corrected well report. Wibby had sent him an email with some of the current wells he had drilled in town with the information that was wrong. The Board would like Wibby to do a letter stating he is in violation of the State regulations with incorrect information on the reports. Wibby will do up a letter and send along to the Board for their input before she sends to Gap Mountain.

5:10 p.m. In open meeting Andrea made a motion to go into Executive Session to discuss imminently threatened or otherwise demonstrably likely litigation to a proposed tobacco regulation: Ed seconded the motion. Peter Munro, aye, Ed Simoncini, aye, Andrea Crete, aye. Andrea declared the Board would reconvene in open session.

TOWN OF WESTMINSTER
11 South Street
WESTMINSTER, MASSACHUSETTS 01473
(978) 874-7409 • Fax (978) 874-7460
BOARD OF HEALTH

5:37 p.m. Ed made a motion to close Executive Session: Peter Munro aye, Andrea Crete aye, Ed Simoncini aye. The Board reconvened in opening meeting at 5:37 p.m.

5:37 p.m. The Board discussed the steps to take prior to the Public Hearing. Businesses that sell tobacco products will be given a copy of the Draft Regulations in person by Joan Hamlett and will be notified of when the Public Meeting will be held. Wibby will notify the school nurses. DJ will get the draft ready so Town Counsel will have a chance to review it. Cheryl will update town Counsel with where we are with the procedure. A letter will go to the Selectmen. DJ and Cheryl will be glad to meet with the Selectmen to answer any questions or concerns. Wibby will be the one taking all calls for the Board on the upcoming meeting. The Board asked her to be the spokesperson for media. A letter with Talking Points will be drafted on what to say. The Board thanked Cheryl, DJ, Jenn, and Joan for coming to the meeting.

6:15 p.m. Peter made a motion to adjourn, Ed seconded the motion. All were in favor

Andrea Crete	Peter Munro	Edward Simoncini
Andrea Crete, Chairman	Peter Munro	Edward Simoncini

Minutes were signed by Board members on 11/19/20414   Original signatures on file in BOH office.

Attachments:
Form 9A 100 Town Farm Road
100 Town Farm Road septic system plan
Public Body Checklist for Entering into Executive Session
