# Supplementary material for: Tobacco industry and public health responses to state and local efforts to end tobacco sales from 1969-2020
Source: PLoS One. 2020 May 22;15(5):e0233417. doi: 10.1371/journal.pone.0233417 (PMC7244130; doi:10.1371/journal.pone.0233417)
Supplement: Attachment — Submitted filename: Response to reviewers_Final.docx [file pone.0233417.s004.docx]

Response to Reviewers

**Editor’s comments**

There are two things that I think are especially important for a revision (also highlighted by the referee reports). First, there needs to be more information on the search strategy and what was actually done. Second, the authors need to do a lot more on the analysis of the data -- what is it about this instance that the ban went through while in the others it did not? What kinds of initiatives by the state draws what kinds of an industry response? Are there correlations in the characteristics that help one understand the strategies adopted by the industry. More statistical rigor will help in making a stronger case to understand the specific question posed.

*We have revised the paper substantially, with clearly stated research questions. We have also added information on several additional California cities that have recently proposed tobacco sales bans (Hermosa Beach and Manhattan Beach) and on one state (North Dakota) whose 2003 tobacco sales ban came to our attention as we reviewed additional materials related to a 1990 proposal to ban sales. We have added new tables and re-organized our data in a way that allows us to highlight similarities in the tobacco industry’s response to tobacco sales bans versus other tobacco control policies, and to identify changes in public health organizations’ attitudes towards tobacco sales bans. We have also provided more information on our search strategy, as suggested by both the editor and reviewers, including details on keywords used and exclusion criteria. We provide more details on these changes in our response to both reviewers below. As noted below by both reviewers, statistical analysis is not applicable to this archival study so we interpreted the comment about statistical rigor in terms of qualitative methodological rigor.*

Reviewers' comments:

Reviewers’ Responses to Questions

**Comments to the Author**

1. Is the manuscript technically sound, and do the data support the conclusions?

The manuscript must describe a technically sound piece of scientific research with data that supports the conclusions. Experiments must have been conducted rigorously, with appropriate controls, replication, and sample sizes. The conclusions must be drawn appropriately based on the data presented.

Reviewer #1: Partly

Reviewer #2: Yes

2. Has the statistical analysis been performed appropriately and rigorously?

Reviewer #1: N/A

Reviewer #2: N/A

3. Have the authors made all data underlying the findings in their manuscript fully available?

The [PLOS Data policy](https://urldefense.proofpoint.com/v2/url?u=http-3A__www.plosone.org_static_policies.action-23sharing&d=DwMGaQ&c=iORugZls2LlYyCAZRB3XLg&r=MgjqeT2ZlKiFLLlrNLqflBtXyGpxxwfTHtXtX7UqHlQ&m=JCcb4b1EJJme3Go04S9Q6V2QuP1gcb9pRLuSpFi9HPs&s=DSSXKzaMp1ofHugNkE8sTu3JBOehSYLjOydnr55hiKk&e=) requires authors to make all data underlying the findings described in their manuscript fully available without restriction, with rare exception (please refer to the Data Availability Statement in the manuscript PDF file). The data should be provided as part of the manuscript or its supporting information, or deposited to a public repository. For example, in addition to summary statistics, the data points behind means, medians and variance measures should be available. If there are restrictions on publicly sharing data—e.g. participant privacy or use of data from a third party—those must be specified.

Reviewer #1: Yes

Reviewer #2: Yes

4. Is the manuscript presented in an intelligible fashion and written in standard English?

PLOS ONE does not copyedit accepted manuscripts, so the language in submitted articles must be clear, correct, and unambiguous. Any typographical or grammatical errors should be corrected at revision, so please note any specific errors here.

Reviewer #1: Yes

Reviewer #2: Yes

5. Review Comments to the Author

Please use the space provided to explain your answers to the questions above. You may also include additional comments for the author, including concerns about dual publication, research ethics, or publication ethics. (Please upload your review as an attachment if it exceeds 20,000 characters)

Reviewer #1: Main Comments

Thank you for the opportunity to review this paper which analyses efforts, over many decades, to adopt bans on the sale of cigarettes in the US, and industry efforts to oppose such bans. Fuller understanding of tobacco industry efforts to oppose, undermine and even prevent the adoption of effective tobacco control measures in different policy contexts is an important knowledge contribution. On this basis, the aim of this article is important. However, I am not convinced of the extent to which this aim has been achieved by the authors in two respects. First, the authors state that there has been no detailed analysis to date of how the industry “has anticipated and reacted to efforts to end tobacco sales.” This is a statement of the originality of the subject matter and I would have liked to see a tight brief review first of what we already do know about industry opposition to tobacco control measures in the US, which measures, what their tactics have been, and with what effect. This would provide important context for the analysis of sales bans that follows.

*We have revised the introduction to give the paper more structure, including a brief review of previous research on the tactics and arguments that the tobacco industry has used to oppose tobacco control measures in the US and internationally. This research also informs our analysis.*

Second, the authors need to explain the significance of understanding industry strategies regarding sales bans beyond that it has not been studied before. Is this to confirm more of the same, regarding industry activities, or is there something distinctly valuable about how the industry has behaved regarding sales bans that extends our understanding of industry activities and strategies?

*We now state in the introduction that it is important to understand tobacco industry tactics and arguments in relation to tobacco sales bans in order to inform tobacco control advocates’ responses to future efforts to ban tobacco sales. If the tobacco industry employs tactics and arguments that have not been identified previously, then understanding and preparing for any new tactics and arguments may help tobacco control advocates counter them in future efforts to ban tobacco sales. If the tobacco industry employs the same tactics and arguments that have been identified previously, this knowledge could also inform tobacco control advocates’ approach to future tobacco sales ban proposals, allowing advocates to draw on previous experience in countering tobacco industry opposition to a variety of tobacco control measures, rather than invent a new approach.*

The other main comment that I have about this paper is that the balance between description and analysis needs to shift more towards the latter. While the material presented could make a potentially important contribution to knowledge, as mentioned, the paper is currently largely a description of the information found in internal documents and media sources about what bans were proposed, when and where, and what the industry said to oppose them. There is need for tighter analysis which draws this information together and gives it higher meaning. This would be helped by providing a clearly stated research question at the beginning beyond finding out what the industry has done to oppose sales bans. Can the authors explain the timing of the sales ban policy proposals, how they subsequently faired and why, why the Beverly Hills policy was adopted, and the prospects for future adoption of sales bans as an effective tobacco control measure. This might not all be possible, given the quality of the data available, but delving down more deeply into selected policies is needed to give this paper more analytical substance.

*We have revised the paper so that it has more structure, with a summary of the literature on tobacco industry tactics and arguments used to oppose tobacco control measures, and a set of clearly stated research questions. This structure also informs our results section, shifting the balance away from pure description. As the reviewer acknowledges, however, the available data do not always allow us to provide unequivocal answers about why some policies failed and others were successful.*

Specific Comments
• The Abstract could be revised to sharpen its statement of the key contribution to knowledge of this paper (originality and significance). It could set out, in particular, not only what the paper has done but why it has done this. Is there a specific research question you are seeking to answer? The purpose of the paper is also not clearly stated. Overall, what does the paper contribute as new knowledge of the tobacco industry and its opposition to tobacco control policies, and why is this important to know?

*We have revised the abstract to reflect the rationale for and purpose and contribution of the study, stating that “knowledge of previous efforts to ban tobacco sales in the US, both successful and unsuccessful, may help inform tobacco control advocates' approach to future efforts.” In the introduction to the paper, we have added 7 research questions which provide the framework for our results.*

• It could be made clearer whether the sales bans proposed/adopted refer to all tobacco/nicotine products or only cigarettes and why. What is the scope of the various policies put forth/adopted, and to what extent does this vary across jurisdictions and time? What have been the key debates surrounding bans? This would set up the subsequent analysis more tightly.

*In Table 1, we indicate the scope of each proposed sales ban. We were unable to determine from our various data sources why some policy champions proposed a ban on cigarette sales while others proposed a broader ban on tobacco sales. However, we now note in the results section that the majority of proposals prohibited all tobacco sales, and that most of the cigarette-only proposals occurred between 1970 and 1994. We have also added a paragraph to the discussion exploring this issue.*

• In the Methods, the first sentence may no longer be needed. The substantial number of publications using internal industry documents from this source might make it sufficient to simply name the UCSF Truth Documents collection. The full name of the depository, with a link to it, may just be needed.

*We made the suggested change.*

• More detail is needed in the Methods about how the documents were searched, all the keyword terms used, what the exclusion/inclusion criteria applied were, and how the documents identified were then analysed (e.g. thematically coded). The document analysis is too sparsely described and needs to provide sufficient detail to enable results to be reproducible.

*We have added the requested details to the Methods section.*

• Add “in the US” to end of sentence 1 of Results. There is a universal tone adopted in the paper and, given the global readership of this journal, it is important to acknowledge that this paper is only about the US context.

*Done. We also added “in the US” throughout the paper to try to eliminate the universal tone of the paper.*

• The tobacco industry is described in quite broad terms and referred to as the “industry”. More detailed description, of which companies or industry-related body acted when and where, to oppose the various sales bans, is needed.

*We have created a new table (table 2) that outlines tobacco industry tactics employed to oppose tobacco sales ban proposals; it provides examples of instances where the tobacco industry worked collaboratively, through the Tobacco Institute, and when tobacco companies acted on their own. The table only refers to the “tobacco industry” more broadly when our sources (i.e., media accounts) use that phrasing, without greater specificity.*

• The extent to which industry opposition was a decisive factor in the failure to adopt bans is not clear from the paper’s analysis. This relates to my main comments about description versus analysis. There is no attempt to explain, with evidence, what happened. Some speculation in the Discussion but, to strengthen this paper, this analysis needs to be more central to the research.

*Given gaps in our evidence, we cannot assert with authority the degree to which industry opposition was a decisive factor in the failure to adopt most tobacco sales ban proposals. We have restructured the paper to make it more clear what questions we can answer with the available data.*

• The statement on p. 15, line 153-154, that the industry “consistently mobilized or prepared to mobilize allies and community members to oppose tobacco sales bans” does not seem supported by the evidence presented in the paper. There is a lack of detail about industry activities, beyond the framing of the various proposed policies. Was there an industry strategy across companies for example like Project Whitecoat? What allies were mobilized and what did they do? To what extent was opposition in different cities/towns/states coordinated within the industry? Did industry arguments change over time or in nature? Which were more effective and why? I realize that internal documents may not provide much information but this is needed if one is to describe industry efforts as somehow organized in this way.

*This statement no longer appears in the revised version of the paper. Table 2 provides both an overview of industry activities, and specific examples of how these activities were carried out. Table 3 details specific industry arguments used to oppose tobacco sales bans, and when/where these arguments were employed.*

• The authors describe the adoption of a sales ban in Beverly Hills as a “turning point” in 2019. I wonder if this is premature given that it is only one municipality, no others have so far followed suit, and it may be an aberration especially given its unrepresentative citizenry. Traditionally, the US public has been averse to prohibitions and tend to lean towards libertarian values. The failure to ban guns, for example, is illustrative of this. What is in the pipeline? Are other sales bans being proposed across the state or US?

*As we note in the revised introduction, Beverly Hills has been joined by Manhattan Beach in adopting a tobacco sales ban, and one additional city (Pasadena) is currently considering a ban. In the revised paper, we no longer use the phrase “turning point,” but instead note that it was only recently that any sales ban proposals garnered enough policymaker support to be adopted.*

• There are some passing comparisons of industry arguments with other public policy issues but this is not done systematically. Might comparative analysis allow the authors to draw wider conclusions or lessons going forward?

*As previously mentioned, the revised introduction presents a framework in which to consider tobacco industry arguments used against tobacco sales bans and other tobacco control policies; the results are also presented with this framework in mind.*

• If the authors can make the case that Beverly Hills is,indeed, a turning point, I wondered if this could form the core narrative in this paper to give it more analytical substance. If so, the paper needs to explain more specifically what changed. Was it industry opposition or public health efforts? Or something else. It would be interesting to discuss, in this context, what prevented adoption previously? I would guess that the rapid uptake of e-cigarettes has been a key factor. This is mentioned in passing in the Discussion. Historically, the public health community has not supported sales bans because of the highly addictive nature of nicotine. A ban would have a serious impact on a large number of existing tobacco users. The widespread availability of e-cigarettes creates an alternative (not a safe one albeit) which seems to have softened opposition to banning combustible tobacco products. Given thousands of cases of acute vaping-related lung disease, it would now be interesting to see if support for sales bans wanes.

*It's important to note that California cities’ recent proposals to ban tobacco sales also include bans on electronic cigarettes. Thus, we don’t think that the availability of e-cigarettes played a role in softening public health groups’ opposition to tobacco sales bans. In the revised version of the paper, we discuss, where the evidence permits, why public health groups supported or opposed efforts to ban cigarette or tobacco sales. One pattern that emerges is that, prior to Beverly Hills, public health groups usually opposed sales bans because they argued that there was no evidence that they would be effective. In Beverly Hills, Hermosa Beach, and Manhattan Beach, opposition changed to support, with public health organizations offering a variety reasons for this support, including the likely positive impact of a sales ban on public health.*

• Relatedly, the explanation in the Discussion of the adoption of the Beverly Hills ban, that it “may have been related, in part, to the support of public health groups”, seems a bit broad and speculative. Indeed, this is what one might expect to have answered more clearly in this paper. Rather than the Discussion stating “it could be this” or “it could be that”, the paper needs to pull together the findings more concertedly and state the “so what” takeaway messages from the evidence reviewed.

*We have revised the discussion substantially, so that the sentence in question no longer appears. Nonetheless, it is important to note that some degree of uncertainty remains regarding why sales ban proposals succeeded or failed, given the limited amount of data we were able to collect on many of them.*

• How effective is the ban in Beverly Hills when it is possible to go to a neighbouring municipality to purchase tobacco products? Or internet sales? What can we learn from sales bans regarding supply-side tobacco control measures?

*As the Beverly Hills and Manhattan Beach ordinances will not go into effect until January 2021, we are unable to answer as to how effective the ban is. However, given existing research demonstrating that living a short distance from a tobacco retailer is associated with failed quit attempts, even a marginal increase in travel time/hassle factor may help those trying to quit succeed in overcoming momentary cravings. We note in the conclusion that as the policies in Beverly Hills and Manhattan Beach go into effect, we will be able to gather much-needed evidence of effectiveness.*

• The main results of the paper seem to be that the industry misrepresented bans as unprecedented and framed them in specific terms. The Discussion picks up this point again. This does seem a bit weak and underwhelming as the key finding arising from the research. There is copious evidence that the industry has often not told the truth. What would be far more compelling, as a contribution to knowledge, is details about activities, strategies and tactics. How differently or not did the industry behave on sales bans? How effective was the industry? What counterarguments are effective to challenge the industry’s efforts?

*We have revised the results and discussion substantially to offer details on the tobacco industry’s tactics and arguments in relation to tobacco sales bans, and on public health organizations’ rationales for supporting or opposing them.*

Reviewer #2: Review:

Introduction
- Line 49 – perhaps it is worth adding the term ‘licit’ or ‘legal’ in the sentence “… the single most deadly [licit/legal] consumer product.” The fact that tobacco is legal seems to be the punctuation to this point.

*The framing of tobacco as “legal” is a tobacco industry argument (as we point out in Table 3) that is often used to obfuscate the issue of whether it should be legal to manufacture and sell deadly products. We prefer not to reify this industry framing. Any product is “legal” only insofar as policy authorities permit or prohibit its sale.*

- Line 50 – I think this is a clear and accurate point pertaining to the health implications of tobacco leading to considerations of banning cigarettes. I wonder if it is worth adding that this movement is also tied up in competing/conflicting values of individualism, hygiene (the puritan movement), the proper place of government in society, etc. This is a minor point, but may cast this history in a broader context beyond simply a technical and linear relationship between health risk and public policy.

*We take your point; given space limitations, we changed the sentence so that it now refers to moral as well as health concerns influencing states’ decision to ban cigarette or tobacco sales in the late 19^th^ and early 20^th^ centuries.*

- Very clear and concise introduction to the topic of the paper. Compelling points about the need to examine the industry response to proposals and actions to ban the sale of tobacco.

*Thank you – although we have now revised the introduction to provide more of a framework for our results and specify our research questions.*

Methods
- I think it will be important to provide more information on the search strategy to retrieve the 110 relevant documents. How did the authors ensure that all relevant documents were identified? Do the authors think that it is possible that any documents were missed? If so, why, and with what implications for the analysis?

*We now provide more information on the search strategy in the methods section. It is highly likely that we missed some relevant documents. We address this point in the limitations paragraph in the discussion, noting that our tally of sales ban proposals is likely a conservative estimate given the likelihood of missing documents.*

- The news media search strategy is well described and provides a clear picture of how the authors approached this search. The strategy of identifying cities and states where tobacco bans were brought forward seems like it could be utilized in the industry documents as well, using the cities and states as search terms in combination with ‘sales ban’ and any other relevant substantive terms. Did the authors utilize this approach in the search strategy for the industry documents?

*We did use the same approach in both the media and industry documents searches; we have clarified that in the revised methods section.*

- Lines 109-111 – please provide more information on the number of ‘selected key documents’ reviewed by the second author? How were these documents selected? How was disagreement resolved? And prior to this, what was the second author examining in these documents? What type of verification was sought? Finally, please explain what is meant by ‘case study’? Perhaps this could also be clarified by the presentation of a more pointed research question in the introduction section. In other words, was this a case study of industry responses, industry action, how opposition was mobilized, who was engaged in this opposition, what types of arguments were brought forward by industry opponents, etc.?

*We have added information on the number of documents reviewed by both authors, and why they were selected. (We also added a series of research questions to the introduction). Finally, we revised our description of our methods, no longer using the term “case study,” but instead describing our process as involving the creation of a timeline of events, with our findings organized in relation to our research questions.*

Results
- Line 134 – the authors note that the industry ‘apparently prepared a 6-page document entitled …’ Is there a reason for the term ‘apparently’? Were the authors unable to obtain this document? If not, I think it would be beneficial to mention how the knowledge of this document was gained.

*We have revised this sentence to better explain that this document lacks a clear author: The [Tobacco] Institute may also have been responsible for preparing a document (with no assigned author) entitled “In Opposition to a Cigarette Sales Ban” outlining, over 6 pages, why Salem’s “ill-founded” proposal should be rejected.*

- The results are quite interesting. The two tables are helpful to gain an overview of the key proposal and the specific opposition to these proposals of banning tobacco. As I was reading the results I kept thinking about who it was bringing forward the proposal to ban tobacco in the various localities. As the authors find, the tobacco control community wasn’t too engaged in supporting or opposing these proposals. It would enrich the findings to add some discussion of the motivations or stated rationale by those proposing a ban on tobacco. Without this context the paper does provide interesting insights into the way that the industry opposed such measures, however it is quite similar to opposition to other tobacco control measures. The novelty is perhaps in the messaging of such a ban being ‘radical’ or ‘fanatical’, although even this type of messaging is seen in other novel measures such as standardized tobacco packaging or flavouring bans. E.g.

Lencucha, R., & de Lima Pontes, C. (2018). The context and quality of evidence used by tobacco interests to oppose ANVISA’s 2012 regulations in Brazil. Global public health, 13(9), 1204-1215.

Lencucha, R., Drope, J., & Labonte, R. (2016). Rhetoric and the law, or the law of rhetoric: how countries oppose novel tobacco control measures at the World Trade Organization. Social Science & Medicine, 164, 100-107.

Among others

*Thank you for providing these two very helpful references, which informed how we restructured the paper to better situate it in relation to previous work on tobacco industry tactics and arguments. We also added a column to Table 1 that summarizes the rationale given for each tobacco sales ban proposal.*

Discussion
- The discussion is well-written and compelling. My only suggestion is that the authors go a bit deeper into the hypothesis that the involvement of tobacco control-public health organizations may have been the factor that led to the passing of the sales ban in Beverly Hills. Is there any evidence of this involvement tipping the scales in the process of adopting the legislation?

*We have revised the results section so that it provides more detail about public health organizations’ activities in Beverly Hills, Hermosa Beach, and Manhattan Beach; we have also revised the discussion, providing more analysis of possible reasons for changes in public health organizations’ support for tobacco sales bans (although we are unable to assert with certainty why public health groups changed their approach). Unfortunately, the data we have do not allow us to determine whether the support of public health groups played the decisive role in tipping the scales towards successful adoption of policies in California in 2019 and 2020.*

- Similar to my final comment on the results section, I think it is important for the authors to add more information about the way that that tobacco ban was framed and argued for by those bringing forward the proposal. It is both academically interesting and practically important to know how such a novel bill was framing and supported both in terms of evidence and argumentation. I would like to see this added to the discussion.

*In the results section, we added a column to Table 1 that indicates the rationale given for each tobacco sales ban proposal by those who brought it forward. We also added a table (Table 4) outlining the public health groups’ support for or opposition to tobacco sales bans, rationale/arguments, and activities. In the discussion, we note that these bans were typically justified in the name of public health, much as other tobacco control measures were justified, and did not rely on a unique set of arguments to justify them.*

Overall, this is an interesting, timely and well-research paper. I think it will clearly contribute to the literature on industry approaches to novel tobacco control measures.

*Thank you!*
